# Fluid Shear Stress Drives Endothelial Glycolytic Reprogramming via an AIBP/VEGF Axis

**DOI:** 10.3390/biology15181650

**Published:** 2026-09-18

**Authors:** Linhua Zhou, Xiaowu He, Yaowu Liu, Yanpeng Wei, Yiting Yang, Qi Zhang

**Affiliations:** 1 Orient Vascular Innovation Institute, University of Shanghai for Science and Technology, Shanghai 200093, China; 2 Department of Clinical Neurology Medical Center, 411 Hospital, Shanghai University, Shanghai 200081, Chinaliuyaowu1227@126.com (Y.L.);

**Keywords:** AIBP, shear stress, VEGF, glycolysis, endothelial cells

## Abstract

Hemodynamic shear stress is a critical mechanical determinant of endothelial homeostasis, yet the mechanosensitive pathways governing metabolic reprogramming remain incompletely understood. Apolipoprotein A-I binding protein (AIBP) regulates cholesterol metabolism, but its role in endothelial mechanotransduction is unknown. Here, we investigated how shear stress modulates AIBP expression to direct human brain microvascular endothelial cell (HBMEC) function via vascular endothelial growth factor (VEGF)-dependent glycolytic reprogramming. HBMECs were subjected to laminar shear stress (1 Pa) with or without the mechanosensitive channel inhibitor GsMTx4. We utilized loss-of-function approaches (shRNA targeting AIBP and VEGF), metabolic assays (metabolite quantification, Western blotting of glycolytic enzymes), and functional assays (EdU proliferation, Transwell migration, tube formation, and apoptosis analysis). Laminar shear stress significantly downregulated AIBP levels via mechanosensitive channel activation. Downregulation or genetic silencing of AIBP stabilized HIF-1α, relieving constitutive repression of VEGF and activating downstream Akt and ERK1/2 signaling. This axis drove glycolytic reprogramming—evidenced by coordinated upregulation of GLUT1, HK2, PFK-A, PKM2, PTBP1, and LDHA, and accumulation of L(+)-lactate and pyruvate—which enhanced proliferation, migration, and tube formation while attenuating apoptosis. Pharmacological inhibition with GsMTx4 prevented shear-induced AIBP downregulation and subsequent metabolic activation. Crucially, simultaneous knockdown of VEGF abolished the glycolytic and phenotypic shifts induced by AIBP deficiency. AIBP functions as a novel mechanosensitive effector in endothelial cells. Shear stress-induced, channel-dependent downregulation of AIBP orchestrates endothelial activation strictly through VEGF-mediated glycolytic reprogramming, identifying a key mechano-metabolic coupling mechanism in vascular regulation.

## Simple Summary

Our blood vessels are constantly exposed to fluid shear stress caused by blood flow. This study investigates how this physical force affects the energy metabolism of endothelial cells lining the vessel wall. We discovered that shear stress drives a metabolic switch toward glycolysis through a pathway involving AIBP and VEGF. This change helps endothelial cells adapt to blood flow and maintain vascular health. Our findings may provide new targets for treating cardiovascular diseases such as atherosclerosis.

## 1. Introduction

Vascular endothelial cells (ECs) form the inner lining of blood vessels and are continuously exposed to hemodynamic shear stress generated by flowing blood. This dynamic mechanical stimulus is a fundamental determinant of vascular homeostasis, regulating vascular tone, barrier integrity, and inflammatory responses [1,2,3]. Physiological laminar shear stress promotes a quiescent, atheroprotective endothelial phenotype, whereas abnormal or disturbed shear stress, which is often found at arterial bifurcations and curvatures, drives endothelial activation, contributing to the pathogenesis of various vascular diseases such as atherosclerosis, aneurysms, and vascular malformations [4,5,6,7]. To adapt to these dynamic mechanical environments, ECs have evolved sophisticated mechanosensory complexes that convert physical forces into biochemical signals, a process known as mechanotransduction [8,9]. However, the comprehensive network of mechanosensors and their downstream intracellular effectors remains incompletely understood, necessitating the identification of novel mechanotransduction pathways.

Recent advances in vascular biology have highlighted metabolic reprogramming as a central driver, rather than a mere bystander, of endothelial phenotype and function [10,11,12]. Unlike many other cell types that rely primarily on oxidative phosphorylation, healthy ECs are highly glycolytic. During endothelial activation and angiogenesis, ECs further upregulate glycolytic flux to meet the immense energetic and biosynthetic demands required for rapid proliferation, migration, and extracellular matrix remodeling [10,13]. This metabolic shift is characterized by the concerted upregulation of core glycolytic enzymes, including glucose transporter 1 (GLUT1), hexokinase 2 (HK2), 6-phosphofructokinase type A (PFK-A), pyruvate kinase M2 (PKM2), polypyrimidine tract-binding protein 1 (PTBP1), and lactate dehydrogenase A (LDHA) [14]. While the necessity of glycolysis in EC activation is well established, how specific hemodynamic forces couple with these metabolic machineries remains an area of active investigation.

Apolipoprotein A-I binding protein (AIBP), encoded by the NAXE gene, was initially identified as a secreted protein that interacts with apolipoprotein A-I to facilitate cellular cholesterol efflux and modulate lipid raft assembly [15,16]. Emerging evidence suggests that AIBP possesses multifaceted roles beyond basic lipid metabolism, actively participating in the regulation of vascular development, neuroinflammation, and angiogenesis [17]. For instance, AIBP-mediated cholesterol depletion can disrupt the spatial organization of lipid rafts, thereby restraining the hyperactivation of membrane-bound receptors [15,16,17]. Despite these insights, a critical knowledge gap remains: it is entirely unknown whether AIBP acts as a mechanosensitive molecule that responds to hemodynamic shear stress, and if so, how it orchestrates the intricate balance between mechanical stimuli, metabolic reprogramming, and endothelial function.

Vascular endothelial growth factor (VEGF) is the most potent master regulator of angiogenesis and endothelial activation [18,19,20]. The expression and downstream signaling of VEGF are highly sensitive to both biochemical cues and biomechanical forces, such as shear stress [20]. Upon activation, VEGF cascades robustly stimulate endothelial glycolysis by upregulating the aforementioned panel of glycolytic enzymes, thus providing the metabolic foundation for angiogenesis [10,13]. We postulated that AIBP might serve as a crucial molecular brake upstream of VEGF. If hemodynamic shear stress could modulate AIBP expression via specific mechanosensors, it would unveil a novel mechanotransduction axis where physical forces govern endothelial metabolic plasticity through an AIBP–-VEGF–-glycolysis hierarchical network.

In the present study, we hypothesized that physiological laminar shear stress activates upstream mechanosensitive ion channels (such as Piezo1) to downregulate AIBP expression, positioning AIBP as a critical downstream effector that relieves its constitutive repression on VEGF, prompting a massive induction of core glycolytic enzymes (GLUT1, HK2, PFK-A, PKM2, PTBP1, and LDHA), and ultimately driving endothelial cell proliferation, migration, and tube formation. To rigorously test this hypothesis, we employed a highly specific mechanosensitive channel inhibitor, GsMTx4 [21], alongside targeted loss-of-function (shRNA) epistasis models for AIBP and VEGF in human brain microvascular endothelial cells (HBMECs) exposed to well-defined laminar shear stress (1 Pa). By avoiding traditional overexpression artifacts and relying on physiological interventions, our rescue experiments conclusively map a previously unrecognized mechanosensitive channel/AIBP/VEGF/glycolysis signaling axis. This study not only identifies AIBP as a novel shear stress-responsive mechanosensitive downstream effector but also provides profound mechanistic insights into the mechano-metabolic coupling underlying vascular homeostasis, offering potential new therapeutic targets for flow-related vascular pathologies.

## 2. Materials and Methods

### 2.1. Cell Culture

Human brain microvascular endothelial cells (HBMECs) were purchased from ScienCell Research Laboratories (Catalog #1000, Carlsbad, CA, USA). Cells were cultured in Endothelial Cell Medium (ECM, ScienCell, Cat# 1001) supplemented with 5% fetal bovine serum (FBS, ScienCell, Cat# 0025), 1% endothelial cell growth supplement (ECGS, ScienCell, Cat# 1052), and 1% penicillin/streptomycin solution (ScienCell, Cat# 0503). Cells were maintained in a humidified incubator at 37 °C with 5% CO_2_. Cells between passages 3 and 6 were utilized for all subsequent experiments.

### 2.2. Plasmid Construction and Lentiviral Transduction

To establish loss-of-function models, three specific short hairpin RNA (shRNA) sequences targeting human NAXE (encoding AIBP) and VEGFA mRNAs were designed and synthesized by Genomeditech (Shanghai, China), and cloned into the PGMLV-SC5 lentiviral vector containing ZsGreen1 and a puromycin resistance marker. A non-targeting shRNA served as the negative control (NC). shRNA sequences used in this study include: shNC: TTCTCCGAACGTGTCACGT; shAIBP-1: CCGTGGACCAGGAGCTATTTA; shAIBP-2: GCTACGAGCCAACCATCTATT; shAIBP-3: TCCAGCCAGACTTGCTCATAT; shVEGF-1: AGGGCAGAATCATCACGAAGT; shVEGF-2: GCGCAAGAAATCCCGGTATAA; shVEGF-3: GCTTCCTACAGCACAACAAAT. Among them, shAIBP-2 and shVEGF-1 exhibited the highest knockdown efficiencies and were selected for all subsequent experiments (designated as shAIBP and shVEGF, respectively). Lentiviral packaging was performed in HEK-293T cells using the psPAX2 and pMD2.G helper plasmids. Supernatants containing lentiviral particles were harvested 48 h post-transfection and filtered through a 0.45 µm membrane. HBMECs were transduced with the respective lentiviral particles in the presence of 5 µg/mL polybrene (Sigma-Aldrich, St. Louis, MO, USA). After 48 h of transduction, stably integrated cells were selected using 2.5 µg/mL puromycin (Sigma-Aldrich) for 72 h.

### 2.3. Shear Stress Application and Pharmacological Treatment

Fluid shear stress experiments were performed based on well-established mechanobiology protocols. Briefly, HBMECs were seeded at a density of 1 × 10^5^ cells/mL into µ-Slide I 0.4 Luer channel slides (ibidi GmbH, Gräfelfing, Germany). For mechanosensitive channel inhibition experiments, cells were pre-treated with the specific peptide inhibitor GsMTx4 (10 µM, TargetMol, Wellesley Hills, MA, USA) for 1 h prior to mechanical stimulation. Subsequently, a steady physiological laminar shear stress of 1 Pa (10 dyne/cm^2^) was applied using the ibidi Pump System (ibidi GmbH, Germany) for 24 h, with GsMTx4 continuously maintained in the circulating medium. Cells cultured under static conditions in identical slides without flow served as baseline controls.

### 2.4. Quantitative Real-Time PCR (qRT-PCR)

Following the 24-h static or shear stress treatments, cells were harvested and total RNA was extracted using TRIzol reagent (Invitrogen, Carlsbad, CA, USA) according to the manufacturer’s protocol. RNA concentration and purity were measured using a NanoDrop 2000 spectrophotometer (Thermo Fisher Scientific, Waltham, MA, USA). 1 µg of total RNA was reverse-transcribed into cDNA using the PrimeScript RT Reagent Kit (Takara Bio, Shiga, Japan). qRT-PCR was performed on a QuantStudio 5 Real-Time PCR System (Thermo Fisher Scientific) using TB Green Premix Ex Taq II (Takara Bio). The cycling conditions were: 95 °C for 30 s, followed by 40 cycles of 95 °C for 5 s and 60 °C for 34 s. GAPDH served as the internal reference. Relative gene expression levels were calculated using the 2^−ΔΔCt^ method. The primer sequences used are as follows: NAXE (AIBP): Forward: 5′-TGCTGTGATGGGCATTCTGA-3′, Reverse: 5′-TGCAGAGATGAAGTTGGCGT-3′; VEGFA: Forward: 5′-AGGGCAGAATCATCACGAAGT-3′, Reverse: 5′-AGGGTCTCGATTGGATGGCA-3′; GAPDH: Forward: 5′-GGAGCGAGATCCCTCCAAAAT-3′, Reverse: 5′-GGCTGTTGTCATACTTCTCATGG-3′.

### 2.5. Western Blot Analysis

After the respective treatments, cells were washed with ice-cold PBS and total cellular proteins were extracted using RIPA lysis buffer (Beyotime Biotechnology, Shanghai, China) supplemented with a protease and phosphatase inhibitor cocktail. Protein concentrations were determined using a BCA Protein Assay Kit (Beyotime). Equal amounts of protein (30 µg/lane) were separated by 10% or 12% SDS-PAGE and electrophoretically transferred onto 0.22 µm PVDF membranes (Millipore, Billerica, MA, USA). Membranes were blocked with 5% non-fat milk in TBST for 1 h at room temperature, followed by overnight incubation at 4 °C with the following primary antibodies: anti-AIBP (Abclonal, Wuhan, China; A15952, 1:1000), anti-VEGF (Abcam, Cambridge, UK; ab46154, 1:1000), anti-GLUT1 (Proteintech, Wuhan, China; 21829-1-AP, 1:1000), anti-HK2 (Proteintech, 22029-1-AP, 1:1000), anti-PFK-A (Proteintech, 55028-1-AP, 1:1000), anti-PKM2 (Cell Signaling Technology, Danvers, MA, USA; #4053, 1:1000), anti-PTBP1 (Proteintech, 12582-1-AP, 1:1000), anti-AKT (Proteintech, 10176-2-AP, 1:1000), anti-p-AKT (Proteintech, 28731-1-AP, 1:1000), anti-HIF-1α (Proteintech, 66730-1-AP, 1:1000), anti-Erk1/2 (Proteintech, 11257-1-AP, 1:1000), anti-pErk1/2 (Proteintech, 80031-1-AP, 1:1000), anti-LDHA (Proteintech, 19987-1-AP, 1:1000), and anti-GAPDH (Proteintech, 60004-1-Ig, 1:5000). After washing with TBST three times, the membranes were incubated with HRP-conjugated goat anti-rabbit or anti-mouse IgG secondary antibodies (Cell Signaling Technology, 1:5000) for 1 h at room temperature. Protein bands were visualized using an Enhanced Chemiluminescence (ECL) kit (Millipore) and captured using a ChemiDoc XRS+ System (Bio-Rad, Hercules, CA, USA). Band intensities were quantified using ImageJ software (NIH version 1.46, Bethesda, MD, USA).

### 2.6. Cell Viability and Proliferation Assays

Cell viability was evaluated using the Cell Counting Kit-8 (CCK-8, Beyotime, Cat# C0038). Briefly, following the 24-h static or shear stress treatments, HBMECs were harvested via trypsinization, resuspended, and re-seeded in 96-well plates (5 × 10^3^ cells/well). HBMECs were seeded in 96-well plates (5 × 10^3^ cells/well) and cultured for the indicated consecutive days (0, 1, 2, and 3 days). At each time point, 10 μL of CCK-8 solution was added to each well and incubated at 37 °C for 2 h in the dark. Absorbance at 450 nm was measured using a Synergy H1 microplate reader (BioTek, Winooski, VT, USA). Cell proliferation was further determined using an EdU Cell Proliferation Kit (Beyotime, Cat# C0071S). Cells were incubated with 10 µM EdU for 4 h, fixed with 4% paraformaldehyde, permeabilized with 0.5% Triton X-100, and stained with Click-iT reaction cocktail. Nuclei were counterstained with Hoechst 33342. Images were acquired using an Olympus CKX53 inverted fluorescence microscope (Olympus, Tokyo, Japan).

### 2.7. Migration and Tube Formation Assays

Endothelial cell migration was assessed using 24-well Transwell chambers with 8 µm pore polycarbonate membranes (Corning, NY, USA). After completion of the designated treatments, HBMECs were trypsinized, counted, and suspended in 200 µL serum-free medium (5 × 10^4^ cells) before being added to the upper chamber, while 600 µL of complete medium containing 10% FBS was added to the lower chamber as a chemoattractant. After 24 h at 37 °C, cells on the upper surface were removed, and the migrated cells on the lower surface were fixed and stained with 0.1% crystal violet. For tube formation assays, 96-well plates were pre-coated with 50 µL/well of growth factor-reduced Matrigel (Corning, Cat# 356231) and polymerized at 37 °C for 30 min. The pre-treated HBMECs (3 × 10^4^ cells/well) were harvested, suspended in serum-free medium, and seeded onto the Matrigel. After 6 h, capillary-like tube networks were photographed using the Olympus CKX53 microscope and analyzed.

### 2.8. Flow Cytometry for Apoptosis

Apoptosis was quantified using the Annexin V-FITC/PI Dual Staining Kit (Beyotime, Cat# C1062L). Following treatments, cells were harvested via EDTA-free trypsinization, washed with cold PBS, and resuspended in 1× Binding Buffer. Cells were then incubated with 5 µL Annexin V-FITC and 5 µL Propidium Iodide (PI) for 15 min at room temperature in the dark. Samples were analyzed within 1 h using a FACSCanto II flow cytometer (BD Biosciences, San Jose, CA, USA). Data were analyzed using FlowJo software (v10.8, FlowJo LLC, Ashland, OR, USA).

### 2.9. Intracellular Metabolite Detection

To assess glycolytic and metabolic reprogramming, cells were rapidly harvested following the static or shear stress treatments, and the intracellular levels of key metabolites were measured using commercial colorimetric assay kits according to the manufacturer’s instructions. Specifically, L(+)-Lactate (Cat# S0208S), Pyruvate (Cat# S0299S), Total Nitric Oxide (NO) (Cat# S0024), and Total Cholesterol (Cat# S0211S) kits were all purchased from Beyotime Biotechnology (Shanghai, China). Absorbance readings were normalized to the corresponding total protein concentration for each sample.

### 2.10. Statistical Analysis

All quantitative data are expressed as the mean ± standard error of the mean (SEM) from at least three independent experiments. Statistical analyses were performed using GraphPad Prism 9.0 software (GraphPad Software, San Diego, CA, USA). Two-group comparisons were performed using an unpaired Student’s *t*-test. Multigroup comparisons were conducted using one-way analysis of variance (ANOVA) followed by Tukey’s post hoc test. A *p*-value < 0.05 was considered statistically significant.

## 3. Results

### 3.1. Shear Stress Downregulates AIBP to Promote VEGF Expression via Mechanosensitive Channels

**Figure 1:**
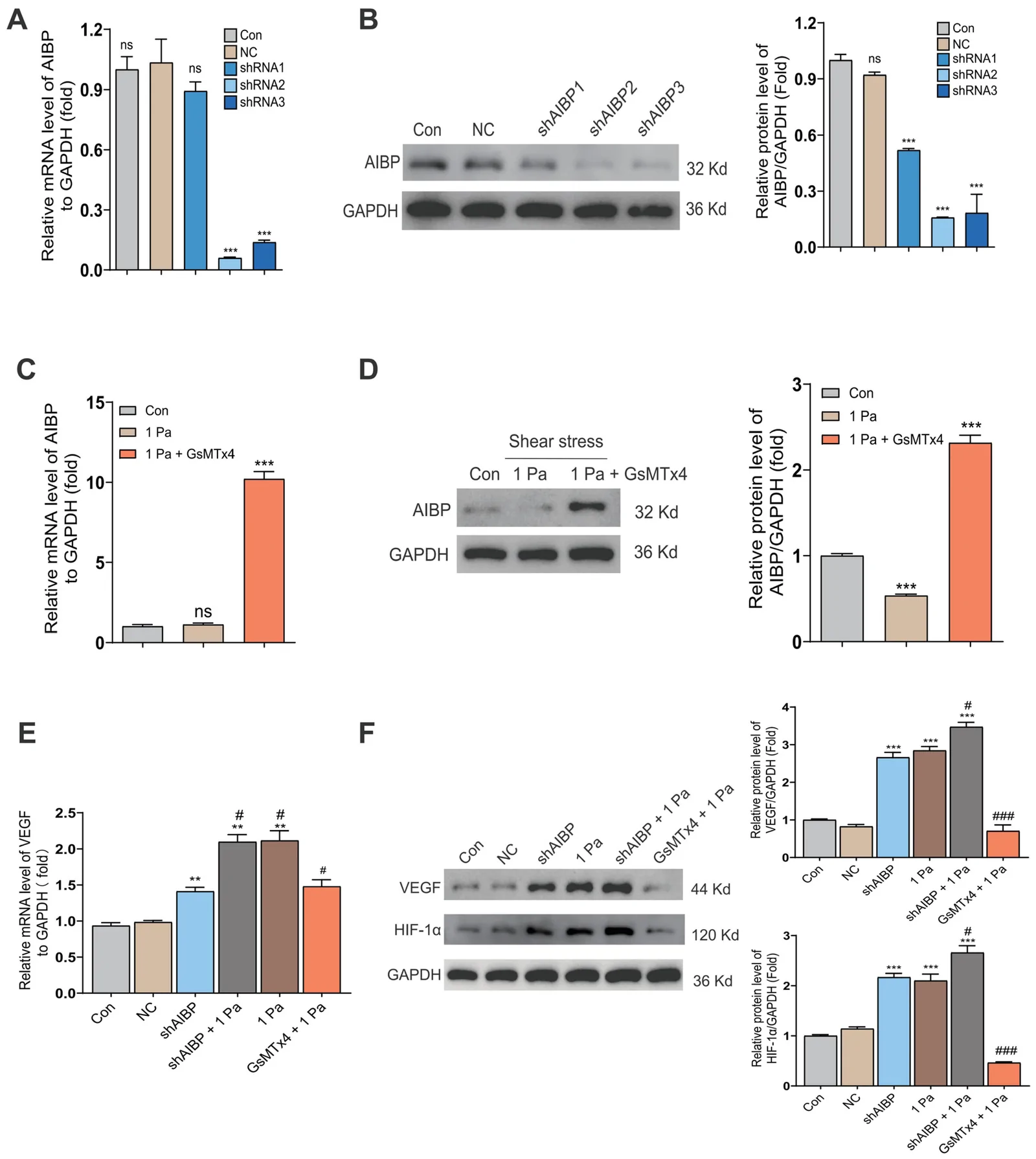
Mechanosensitive channel inhibition prevents shear stress-induced AIBP downregulation and subsequent VEGF expression in HBMECs. (**A**,**B**) Relative AIBP mRNA levels and protein levels were measured by qPCR (**A**) and Western blot (**B**), respectively. Screening of knockdown efficiency in HBMECs transfected with negative control (shNC) or three independent shRNAs (shAIBP-1, shAIBP-2, shAIBP-3). shAIBP-2 (designated as shAIBP) demonstrated the highest silencing efficiency and was designated as shAIBP for all subsequent experiments. (**C**,**D**) Effects of shear stress and mechanosensitive channel inhibition on AIBP expression. HBMECs were exposed to 1 Pa laminar shear stress for 24 h in the presence or absence of the inhibitor GsMTx4. AIBP mRNA (**C**) and protein (**D**) levels were evaluated. GsMTx4 treatment reversed the shear stress-induced downregulation of AIBP. (**E**,**F**) Effects of AIBP knockdown and GsMTx4 treatment on VEGF and HIF-1α expression under static or shear stress (1 Pa) conditions. VEGF mRNA (**E**), and VEGF and HIF-1α protein (**F**) levels were determined. AIBP knockdown further enhanced shear stress–induced VEGF upregulation, whereas GsMTx4 treatment significantly attenuated it. GAPDH served as an internal loading control across all immunoblots. Data are presented as mean ± SEM (N = 3). ** *p* < 0.01, *** *p* < 0.001 vs. Con; # *p* < 0.05, ### *p* < 0.001 vs. 1 Pa; ns, not significant.

To define the role of AIBP in shear stress–mediated endothelial responses, we first generated stable AIBP knockdown HBMEC lines, which were thoroughly validated by qPCR and immunoblotting. Among the three shRNAs tested, shAIBP2 achieved the most robust suppression of AIBP expression and was selected for all subsequent loss-of-function studies (Figure 1A,B; please see supplementary materials for full Western blots). 

Next, we investigated the dynamic response of AIBP to hemodynamic forces. Exposure to physiological laminar shear stress (1 Pa) for 24 h, did not cause a statistically significant change in AIBP mRNA levels (Figure 1C). However, at the protein level, AIBP expression was markedly downregulated (Figure 1D), indicating post-transcriptional or translational regulation. To determine whether this mechanotransduction process depends on mechanosensitive ion channels, cells were treated with the specific inhibitor GsMTx4 during shear stress exposure. Remarkably, GsMTx4 treatment completely reversed the shear stress-induced downregulation of AIBP, profoundly increasing its expression beyond baseline levels (Figure 1C,D).

We further evaluated the downstream impact of this AIBP mechanosensory axis on VEGF, a critical mediator of endothelial activation. Under static conditions, AIBP silencing (shAIBP) was sufficient to significantly upregulate VEGF mRNA and protein expression. Expectedly, laminar shear stress (1 Pa) also induced robust VEGF expression, and this response was further amplified in AIBP-deficient cells exposed to flow (shAIBP + 1 Pa). In stark contrast, pharmacological blockade of mechanosensitive channels with GsMTx4 (GsMTx4 + 1 Pa) effectively preserved AIBP levels and concurrently abrogated the shear stress–induced upregulation of VEGF (Figure 1E,F). Collectively, these data indicate that shear stress activates mechanosensitive channels to downregulate AIBP, thereby relieving its constitutive repression on VEGF expression.

Under static conditions, genetic silencing of AIBP (shAIBP) was sufficient to induce robust VEGF mRNA and protein expression, indicating that basal AIBP exerts constitutive repression over VEGF. Laminar shear stress (1 Pa) alone induced comparable VEGF upregulation, consistent with our observation that 1 Pa shear stress leads to a near-complete downregulation of AIBP. When AIBP-depleted cells were subjected to shear flow (shAIBP+ 1 Pa), VEGF induction was not multiplicatively synergistic over single interventions. This lack of marked additivity indicates that shear stress and AIBP knockdown operate along the same primary mechanistic cascade—namely, the relief of AIBP-dependent VEGF repression. Once AIBP is highly suppressed, additional mechanical stimulation produces limited further derepression through this specific node.

Several factors account for the subtle differences in VEGF induction between shear stress and targeted knockdown. While AIBP downregulation is a primary driver, fluid shear stress activates additional mechanotransduction pathways that may converge on the VEGFA promoter alongside HIF-1α stabilization. As shown in Figure 1D, shear stress suppresses AIBP primarily at the protein level, whereas shRNA targets AIBP transcript stability. These distinct modes of inhibition may result in slightly different kinetics of derepression. Co-treatment with GsMTx4 under flow preserved AIBP levels and restored VEGF mRNA to baseline, confirming that mechanosensitive channel activation upstream of AIBP is required for shear-mediated VEGF upregulation.

Furthermore, to elucidate the intermediate signaling between AIBP and VEGF transcription, we evaluated the expression of HIF-1α. Western blot analysis and corresponding densitometric quantifications revealed that either AIBP silencing or shear stress significantly stabilized HIF-1α protein levels. This stabilization was comprehensively reversed by GsMTx4, strictly mirroring the trend of VEGF expression (Figure 1E,F) and providing a potential transcriptional mechanism for the subsequent VEGF upregulation.

### 3.2. Mechanosensitive Channel Activation Promotes Endothelial Activation via AIBP Downregulation

**Figure 2:**
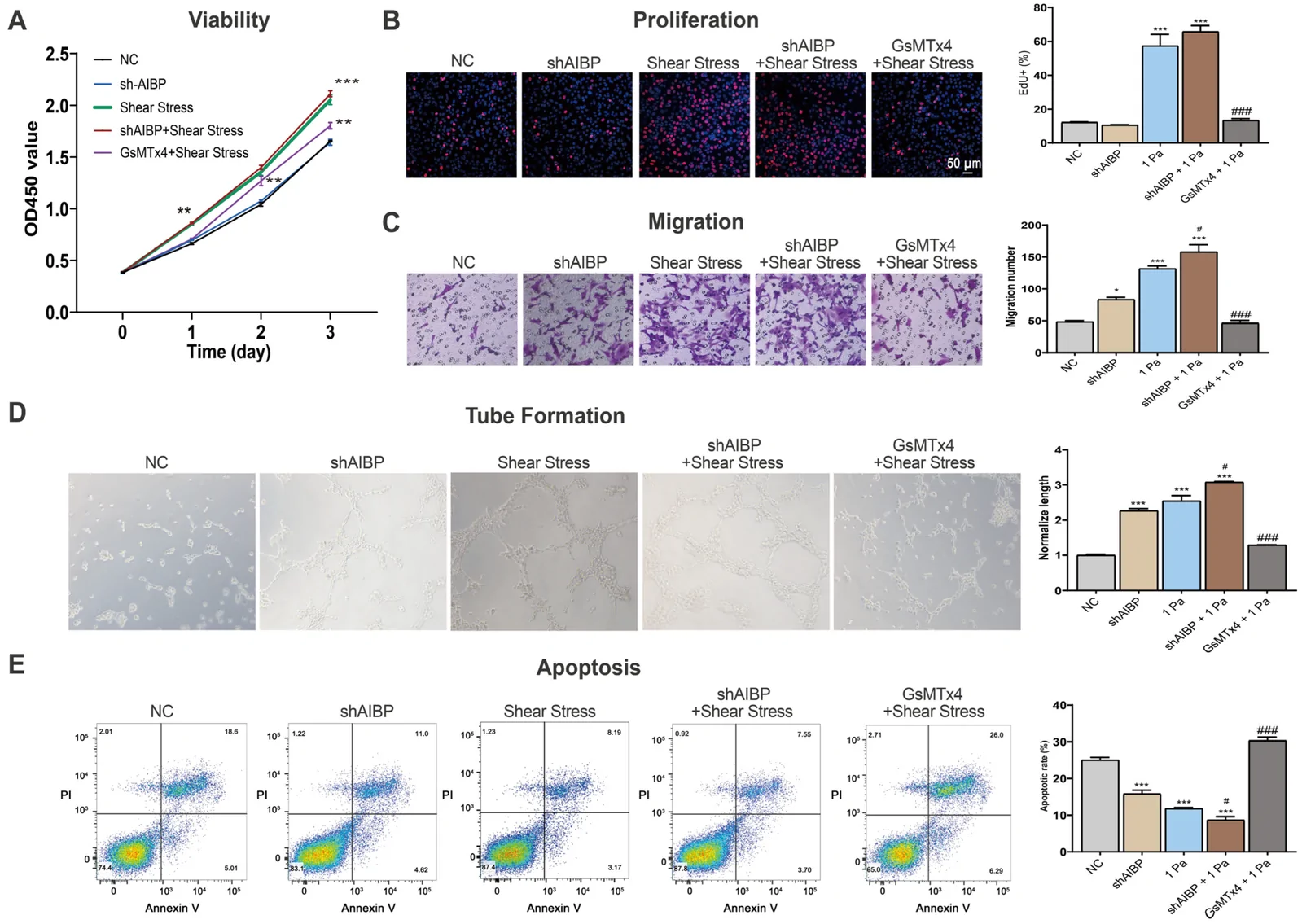
Inhibition of mechanosensitive channels abrogates shear stress-induced endothelial activation. Functional assessments of HBMECs under static conditions, or exposed to 1 Pa laminar shear stress with AIBP knockdown (shAIBP) or GsMTx4 treatment. (**A**) Cell viability evaluated by CCK-8 assay over a 3-day period. (**B**) Cell proliferation determined by EdU incorporation assay. (**C**) Endothelial cell migration assessed by Transwell assay. (**D**) Angiogenic capacity measured by Matrigel tube formation assay. (**E**) Apoptosis quantified by flow cytometry using Annexin V/PI dual staining. AIBP knockdown promoted endothelial viability, proliferation, migration, and tube formation while suppressing apoptosis, and these effects were further potentiated by shear stress (shAIBP + Shear Stress). Conversely, treatment with the mechanosensitive channel inhibitor GsMTx4 (GsMTx4 + Shear Stress) effectively reversed the shear stress-induced pro-angiogenic and pro-survival phenotypes. Data are shown as mean ± SEM (N = 3). Statistical significance as indicated. * *p* < 0.05, ** *p* < 0.01, *** *p* < 0.001 vs. NC; # *p* < 0.05, ### *p* < 0.001 vs. 1 Pa.

To determine the functional consequences of the mechanosensitive channel–AIBP axis, we evaluated essential endothelial activation phenotypes. Under static conditions, AIBP silencing (shAIBP) significantly enhanced migration, and capillary-like tube formation capacities, while markedly reducing cellular apoptosis (Figure 2A–E). Quantitative analyses of these functional assays demonstrated that application of physiological shear stress (1 Pa) alone significantly enhanced endothelial viability, proliferation, migration, and tube formation, while reducing apoptosis (Figure 2A–E). These pro-angiogenic and pro-survival phenotypes were remarkably comparable to, albeit slightly weaker than, the hyperactive responses observed in AIBP-deficient cells exposed to flow (shAIBP + Shear Stress).

Crucially, when mechanosensitive channels were pharmacologically blocked with GsMTx4 during shear stress exposure (GsMTx4 + Shear Stress), the shear stress-induced endothelial activation was significantly attenuated, returning closely to baseline levels. Specifically, GsMTx4 treatment normalized cell viability and proliferation rates (Figure 2A,B), impaired cell migration and tube network formation (Figure 2C,D), and restored the apoptotic rate to baseline levels (Figure 2E). Collectively, these findings demonstrate that shear stress drives endothelial activation by activating mechanosensitive channels, which subsequently relieve AIBP-mediated suppression of angiogenesis and cell survival.

### 3.3. Mechanosensitive Channel Activation Promotes Endothelial Glycolytic Reprogramming via AIBP Downregulation

**Figure 3:**
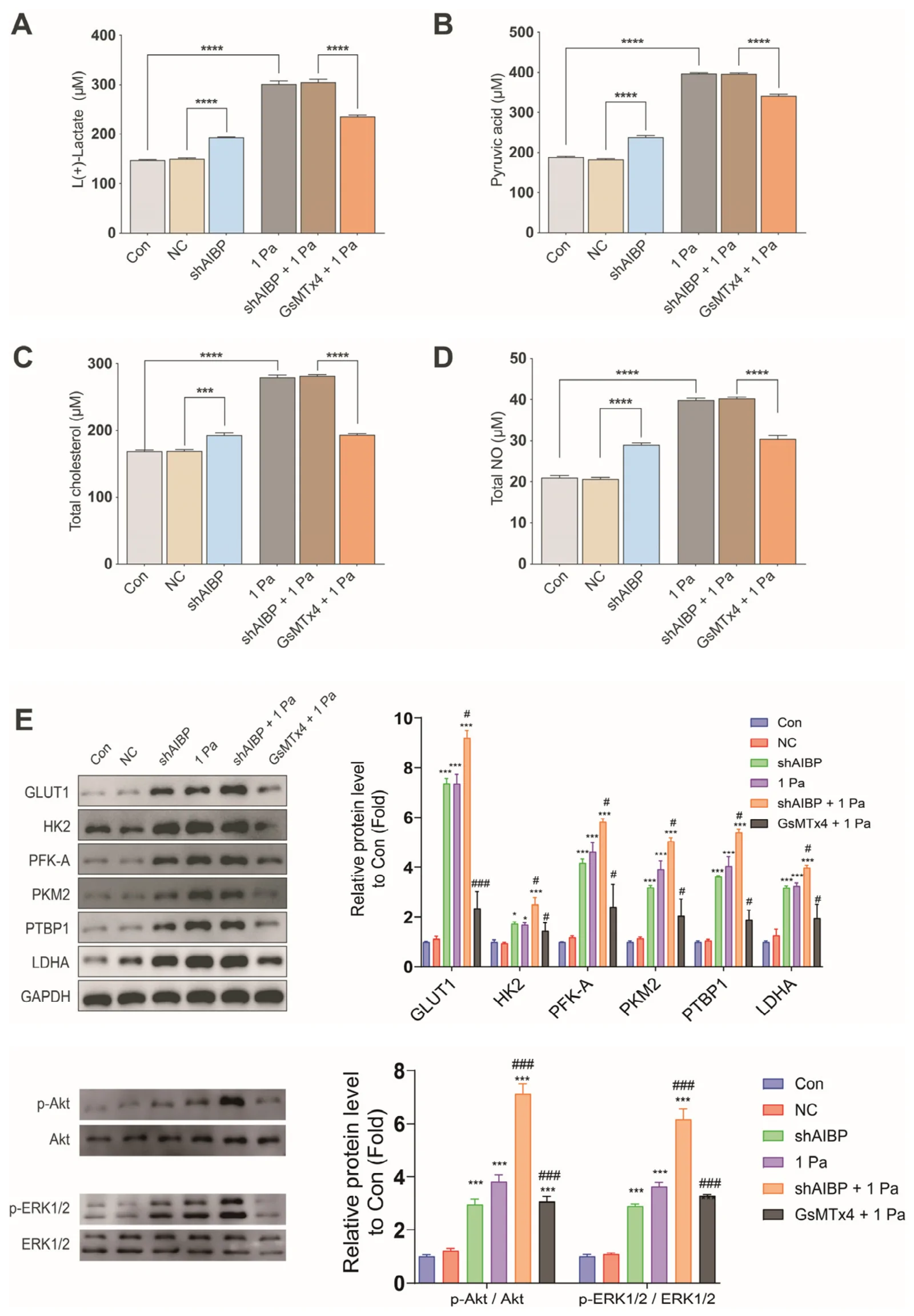
Inhibition of mechanosensitive channels attenuates shear stress-induced glycolytic reprogramming and metabolic shifts. (**A**–**D**) Quantification of intracellular metabolic parameters in HBMECs under static or 1 Pa shear stress conditions, with AIBP knockdown (shAIBP) or GsMTx4 treatment. The measured metabolites include L(+)-lactate production (**A**), pyruvic acid levels (**B**), total cholesterol (**C**), and total nitric oxide (NO) levels (**D**). *** *p* < 0.001, **** *p* < 0.0001. (**E**) Western blot analysis of key glycolytic enzymes, including GLUT1, HK2, PFK-A, PKM2, PTBP1, and LDHA across all experimental groups. GAPDH was used as an internal loading control. Phosphorylation levels of Akt (p-Akt) and ERK1/2 (p-ERK1/2) were also assessed. Shear stress or AIBP knockdown significantly enhanced glycolytic metabolism and increased lipid/NO production, whereas treatment with the mechanosensitive channel inhibitor GsMTx4 (GsMTx4 + 1 Pa) reversed these shear stress-induced metabolic changes. All target protein levels were quantified by densitometry and normalized to GAPDH. Data are presented as mean ± SEM (N = 3). * *p* < 0.05, *** *p* < 0.001 vs. Con; # *p* < 0.05, ### *p* < 0.001 vs. 1 Pa.

The functional changes in endothelial activation were accompanied by a profound metabolic shift. Specifically, under static conditions, AIBP knockdown (shAIBP) led to significantly increased intracellular production of L(+)-lactate and pyruvic acid (Figure 3A,B), elevated total cholesterol and total nitric oxide (NO) levels (Figure 3C,D), and the concerted upregulation of key glycolytic enzymes, including GLUT1, HK2, PFK-A, PKM2, PTBP1, and LDHA (Figure 3E). Exposure to laminar shear stress (1 Pa) induced a similar robust metabolic reprogramming towards glycolysis, which was further amplified by AIBP deficiency (shAIBP + 1 Pa) (Figure 3A–E).

Although shear stress (1 Pa) and AIBP knockdown (shAIBP) induced comparable upregulation of glycolytic enzymes, minor variations in metabolite profiles—specifically total NO and L(+)-lactate—were observed. These differences reflect the broader physiological impact of fluid shear stress. Fluid shear stress directly stimulates eNOS phosphorylation at Ser1177 via mechanical kinase cascades, promoting NO production independently of VEGF transcription. Constitutive shRNA-mediated silencing represents chronic depletion, whereas 24-h shear exposure reflects an active, dynamic adaptive response. Fluid shear stress directly influences mitochondrial bioenergetics and AMPK activation in parallel with the AIBP–VEGF pathway, yielding integrated metabolic outputs.

To further elucidate the downstream signaling cascade linking VEGF to this glycolytic reprogramming, we measured the activation of canonical VEGF downstream pathways. Immunoblotting revealed that both shear stress and AIBP knockdown substantially increased the phosphorylation levels of Akt (p-Akt) and ERK1/2 (p-ERK1/2). This activation of Akt and ERK1/2 signaling paralleled the concerted upregulation of GLUT1, HK2, PFK-A, PKM2, PTBP1, and LDHA, confirming that the mechanosensitive AIBP/VEGF axis drives glycolytic metabolism through these classical kinase cascades.

Pharmacological blockade of mechanosensitive channels with GsMTx4 during shear stress exposure (GsMTx4 + 1 Pa) strongly suppressed the shear stress-induced production of these metabolites and comprehensively attenuated the overexpression of the aforementioned glycolytic enzymes (Figure 3A–E). Collectively, these findings establish that shear stress drives a pro-glycolytic metabolic shift in endothelial cells via a mechanosensitive channel-dependent mechanism that relies on AIBP downregulation, and strengthens the upstream mechanosensitive channel activation required for the shear-driven metabolic shift.

### 3.4. VEGF Is Required for AIBP-Mediated Endothelial Activation

**Figure 4:**
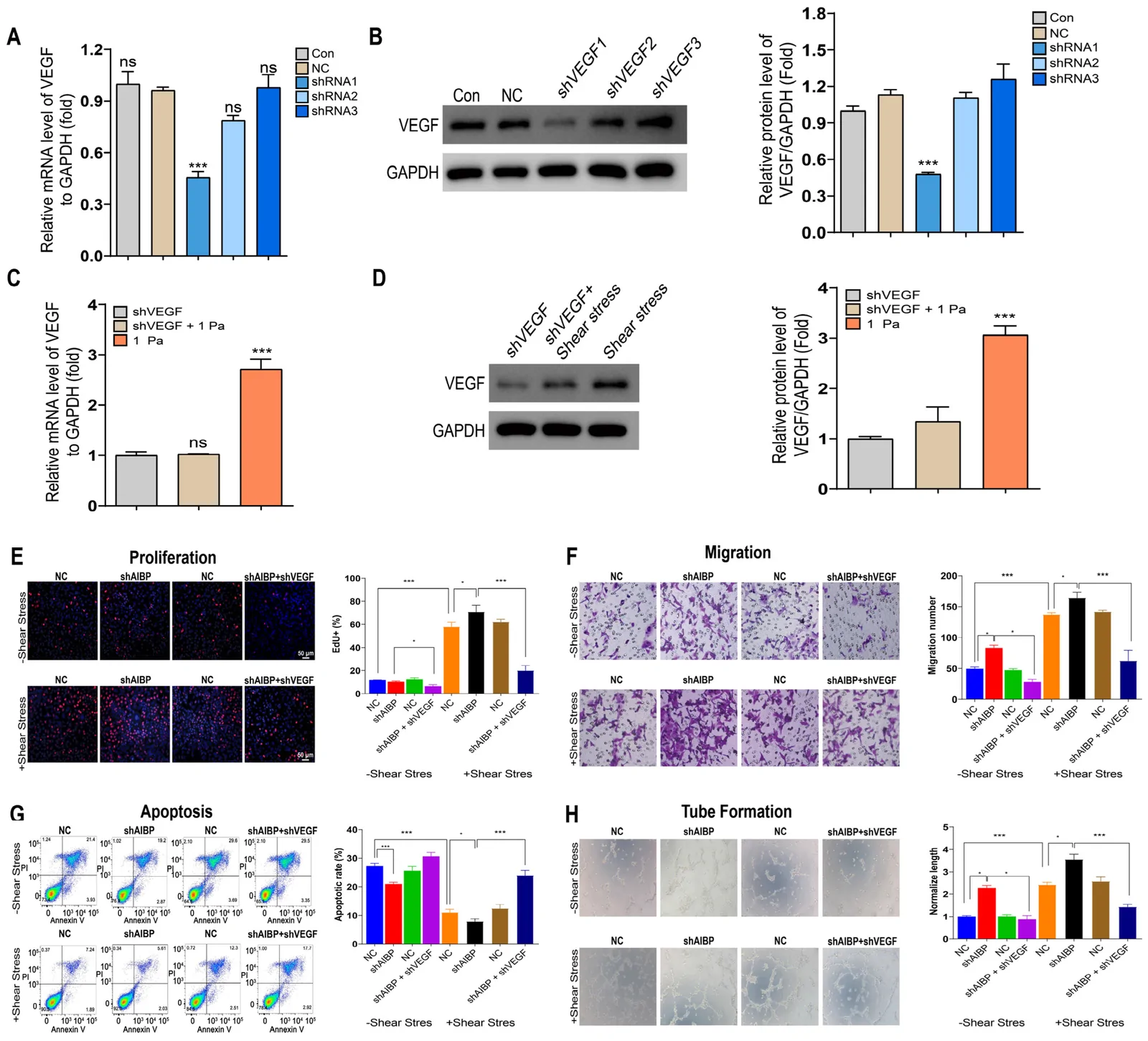
VEGF is required for AIBP-mediated endothelial activation under static and shear stress conditions. (**A**,**B**) VEGF relative mRNA and protein levels were determined by Western blot (**A**) and qPCR (**B**), respectively. Validation of VEGF knockdown efficiency using three candidate constructs (shVEGF-1, shVEGF-2, shVEGF-3). shVEGF-1 was selected for subsequent double-knockdown experiments and is denoted as shVEGF throughout. (**C**,**D**) Effects of VEGF knockdown under shear stress. HBMECs were subjected to 1 Pa shear stress, and the suppressive effect of shVEGF on VEGF mRNA (**C**) and protein (**D**) expression was verified against shear stress-induced upregulation. (**E**–**H**) Rescue experiments assessing endothelial function in cells with AIBP knockdown alone (shAIBP) or double knockdown of AIBP and VEGF (shAIBP + shVEGF). Assays were performed under both static (-Shear Stress) and 1 Pa laminar shear stress (+Shear Stress) conditions. Cellular proliferation (**E**), migration (**F**), apoptosis (**G**), and tube formation capacity (**H**) were evaluated. Simultaneous VEGF silencing completely reversed the pro-angiogenic and anti-apoptotic phenotypes induced by AIBP deficiency across all conditions. GAPDH served as the internal loading control for immunoblots. Data are presented as mean ± SEM (N = 3). * *p* < 0.05, *** *p* < 0.001; ns, not significant.

To definitively establish whether VEGF serves as the essential downstream effector linking the mechanosensitive AIBP axis to endothelial function, we generated and validated stable VEGF knockdown HBMEC models. Among the candidates, shVEGF1 exhibited the highest silencing efficiency and was selected for subsequent experiments (designated as shVEGF) (Figure 4A,B). We further confirmed that this targeted silencing robustly suppressed VEGF expression even under the stimulatory influence of 1 Pa shear stress (Figure 4C,D).

Next, we performed comprehensive epistasis functional assays under both static (-Shear Stress) and flow (+Shear Stress) conditions. Consistent with our previous observations, AIBP deficiency (shAIBP) significantly augmented endothelial proliferation, migration, and tube formation, while concurrently reducing apoptosis (Figure 4E–H). Crucially, simultaneous silencing of VEGF in AIBP-deficient cells (shAIBP + shVEGF) effectively abrogated these hyperactive phenotypes, restoring the pro-angiogenic and pro-survival metrics to near-baseline levels across both static and shear stress conditions (Figure 4E–H). These rescue data conclusively demonstrate that the AIBP-dependent regulation of endothelial activation is fundamentally mediated through its control over VEGF expression.

### 3.5. VEGF Is Required for AIBP-Dependent Endothelial Metabolic Reprogramming

**Figure 5:**
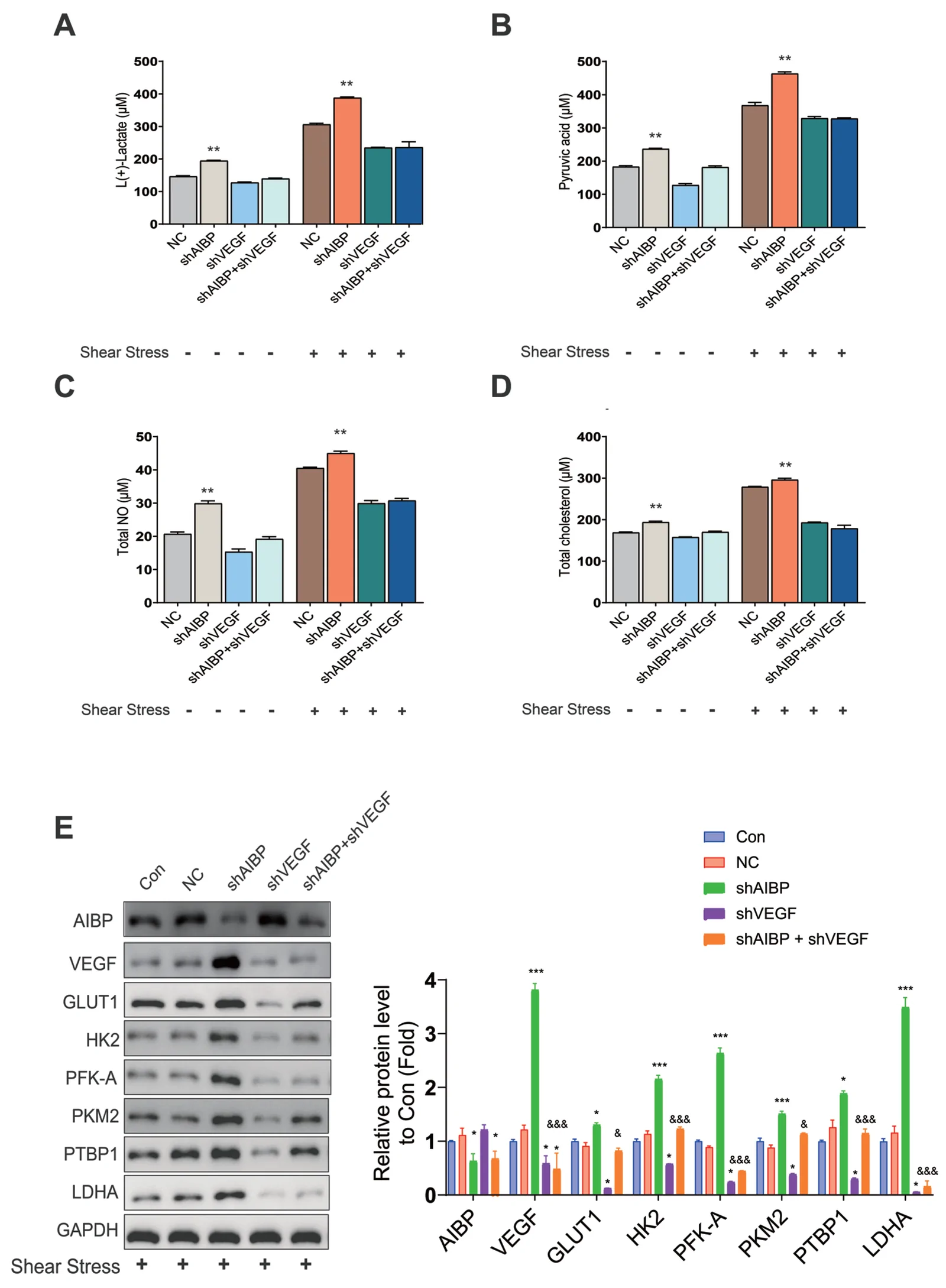
VEGF is required for AIBP-dependent metabolic regulation and glycolytic reprogramming. (**A**–**D**) Quantification of intracellular metabolic parameters in HBMECs subjected to single (shAIBP or shVEGF) or double knockdown (shAIBP + shVEGF) under static (-Shear Stress) or 1 Pa laminar shear stress (+Shear Stress) conditions. The assessed metabolites include L(+)-lactate (**A**), pyruvic acid (**B**), total NO (**C**), and total cholesterol (**D**). VEGF silencing effectively reversed the hypermetabolic state induced by AIBP deficiency under both physiological and mechanostimulatory conditions. (**E**) Western blot analysis of AIBP, VEGF, and core glycolytic enzymes (GLUT1, HK2, PFK-A, PKM2, PTBP1, and LDHA) in cells exposed to shear stress (+). Immunoblotting for AIBP and VEGF confirmed the simultaneous knockdown efficiency in the shAIBP + shVEGF group. The robust upregulation of glycolytic enzymes triggered by AIBP knockdown (shAIBP) was abrogated upon simultaneous VEGF silencing (shAIBP + shVEGF). GAPDH was utilized as an internal loading control. Data are presented as mean ± SEM (N = 3). * *p* < 0.05, ** *p* < 0.01, *** *p* < 0.001 vs. NC; & *p* < 0.05, &&& *p* < 0.001 vs. shAIBP.

Given that VEGF dictates the functional activation of AIBP-deficient endothelial cells, we next sought to determine if this hierarchical axis also governs endothelial metabolic reprogramming. We quantified the intracellular levels of key metabolites in our single and double knockdown models under both static and shear stress conditions. Consistent with our functional data, AIBP depletion (shAIBP) robustly increased the production of L(+)-lactate (Figure 5A), pyruvic acid (Figure 5B), total NO (Figure 5C), and total cholesterol (Figure 5D), irrespective of the presence or absence of shear stress. Crucially, simultaneous knockdown of VEGF (shAIBP + shVEGF) completely reversed these metabolic shifts, normalizing the metabolite levels to baseline across all conditions.

To molecularly confirm this metabolic rescue, we assessed the expression profiles of key glycolytic enzymes under physiological shear stress. Importantly, to confirm the integrity of our epistasis model, we verified the simultaneous knockdown efficiency in the shAIBP + shVEGF group by immunoblotting for both AIBP and VEGF (Figure 5E). While AIBP silencing significantly upregulated the expression of GLUT1, HK2, PFK-A, PKM2, PTBP1, and LDHA, concurrent VEGF depletion (shAIBP + shVEGF) comprehensively abrogated the overexpression of this entire glycolytic panel (Figure 5E). Together, these epistasis experiments position VEGF precisely downstream of AIBP as the principal effector linking mechanotransduction to endothelial metabolic reprogramming and sustained activation, demonstrating that VEGF is the indispensable transducer of this metabolic reprogramming.

## 4. Discussion

The vascular endothelium serves as the critical interface between circulating blood and the vessel wall, constantly decoding physical hemodynamic forces into precise biochemical responses to maintain vascular homeostasis. Aberrations in this mechano-metabolic coupling are fundamental to the onset and progression of vascular pathologies, including atherosclerosis and vascular malformations [4,5,6,7]. In the present study, we identify apolipoprotein A-I binding protein (AIBP) as a previously unrecognized mechanosensitive downstream effector in endothelial mechanotransduction. Our study provides several distinct contributions to the field. First, we demonstrate that laminar shear stress downregulates AIBP expression via mechanosensitive ion channels, identifying AIBP as a novel mechanosensitive downstream effector. Second, we delineate a linear mechano-metabolic axis: Mechanosensitive channel→AIBP→HIF-1α→VEGF→Akt/ERK1/2→Glycolytic reprogramming. Third, we map the complete panel of key glycolytic enzymes (GLUT1, HK2, PFK-A, PKM2, PTBP1, and LDHA) regulated through this pathway. Finally, utilizing double-knockdown (shAIBP + shVEGF) strategies, we establish VEGF as an indispensable downstream functional effector.

Because the specific inhibitor GsMTx4 targets upstream stretch-activated cation channels (such as Piezo1), these membrane channels likely act as the primary, direct mechanosensors. Therefore, the shear stress-induced downregulation of AIBP positions it as a critical downstream node that translates this initial physical detection into sustained biochemical and metabolic responses. The subsequent loss of AIBP releases a molecular brake on VEGF expression, which in turn orchestrates a comprehensive glycolytic reprogramming—evidenced by the concerted upregulation of GLUT1, HK2, PFK-A, PKM2, PTBP1, and LDHA, ultimately driving a hyperactive, pro-angiogenic endothelial phenotype. By employing a stringent double-knockdown (shAIBP + shVEGF) epistasis strategy rather than traditional overexpression models, our findings provide robust, physiologically relevant evidence for the mechanosensitive channel/AIBP/VEGF/glycolysis signaling cascade.

The initial step in our proposed mechanotransduction axis relies on the activation of mechanosensitive ion channels, a process we pharmacologically interrogated using the specific peptide inhibitor GsMTx4 [21]. GsMTx4 is an amphipathic gating modifier that partitions into the lipid bilayer, effectively inhibiting stretch-activated cation channels such as Piezo1 and TRPC1 by altering local membrane mechanics. Our observation that GsMTx4 comprehensively abrogates shear stress-induced AIBP downregulation strongly implicates membrane-bound mechanosensitive channels as the primary sensors upstream of AIBP. While the exact downstream signaling linking these ion channels to AIBP repression requires further elucidation, it is well established that shear stress-induced calcium influx through channels like Piezo1 rapidly activates calcium-dependent kinases and phosphatases (e.g., Calpain, Calcineurin) [2,3]. We hypothesize that such calcium-dependent cascades may either promote the rapid proteasomal degradation of AIBP or recruit transcriptional repressors to the NAXE promoter. Interestingly, pharmacological blockade with GsMTx4 induced a 10-fold increase in NAXE (AIBP) mRNA. The ability of GsMTx4 to preserve AIBP levels under shear stress underscores the dynamic and continuous nature of this mechanosensory regulation. This suggests the existence of a strong compensatory transcriptional mechanism when mechanosensitive ion channels are completely inhibited, warranting future investigation into specific feedback loops.

AIBP has been primarily characterized as a regulator of cellular cholesterol efflux and lipid raft organization [15,16,17]. By modulating membrane cholesterol content, AIBP controls the spatial compartmentalization and signaling efficiency of various membrane-associated receptor complexes [8,9]. Our findings significantly expand this paradigm by identifying AIBP as a crucial suppressor of VEGF expression in endothelial cells (ECs). Under static, quiescent conditions, basal AIBP levels may maintain a lipid raft microenvironment that dampens spontaneous pro-angiogenic signaling. One plausible mechanism is that AIBP regulates the integrity of shear stress–sensing complexes, including integrins, VE-cadherin, and PECAM-1, thereby limiting the activation of downstream signaling pathways (such as PI3K/Akt or HIF-1α) that potently drive VEGFA transcription. Alternatively, AIBP may influence transcriptional control more directly, potentially modulating the activity of shear-sensitive transcription factors such as KLF2 or KLF4 [22]. Regardless of the specific intermediate steps, the complete functional rescue observed in our double-knockdown (shAIBP + shVEGF) model firmly establishes VEGF as the indispensable downstream effector of AIBP, linking the structural and mechanosensory roles of AIBP directly to canonical angiogenic pathways. The concomitant rise in total NO likely reflects shear stress-induced eNOS activation and may also be linked to increased tetrahydrobiopterin (BH4) availability driven by the glycolytic flux, though the precise mechanism warrants further investigation.

Our expanded mechanistic investigations bridge the critical knowledge gap between AIBP lipid raft modulation, VEGF transcription, and subsequent glycolysis. We observed that AIBP downregulation stabilizes HIF-1α, a master transcriptional regulator of VEGFA [23]. It is plausible that AIBP-mediated structural changes in the membrane influence oxygen-independent HIF-1α degradation pathways. Upon VEGF upregulation, we confirmed the robust downstream activation of Akt and ERK1/2 signaling cascades [24,25]. These kinase pathways are well-established drivers of metabolic reprogramming, elegantly explaining the concerted up-regulation of the core glycolytic machinery (GLUT1, HK2, PFK-A, PKM2, PTBP1, and LDHA) observed in our models.

Beyond transcriptional regulation via HIF-1α stabilization, AIBP may exert additional control over VEGF signaling by modulating VEGFR2 activation at the plasma membrane. Given AIBP’s established role in facilitating cholesterol efflux and disrupting lipid rafts [15,16,17], membrane cholesterol depletion typically impedes receptor clustering. Previous studies have demonstrated that intact lipid raft microdomains are required for efficient VEGFR2 dimerization and downstream autophosphorylation [18]. Consequently, the shear stress-induced downregulation of AIBP may not only relieve transcriptional repression of VEGF, but also preserve lipid raft integrity, creating a membrane microenvironment permissive for VEGFR2 dimerization. This dual mechanism—acting both upstream at the transcriptional level (VEGF expression) and downstream at the receptor level (VEGFR2 dimerization)—would synergistically amplify endothelial glycolytic and angiogenic responses. Future investigations employing proximity ligation assays (PLA) and membrane fractionation will be valuable to delineate the precise contributions of these two regulatory layers.

Perhaps the most striking physiological consequence of the AIBP–VEGF axis is its profound impact on endothelial metabolic reprogramming. It is now widely recognized that metabolism is not merely a consequence of endothelial activation, but rather a primary driving force [12,26,27,28]. Unlike most healthy cells, ECs rely heavily on glycolysis, even in oxygen-rich environments, to rapidly generate ATP and essential biosynthetic precursors required for cell division and migration [13]. Our study reveals that the AIBP–VEGF axis regulates not just a single metabolic enzyme, but rather a comprehensive glycolytic panel. Specifically, the concerted upregulation of GLUT1, HK2, PFK-A, PKM2, PTBP1, and LDHA following AIBP depletion signifies a total commitment to the glycolytic phenotype. Notably, the upregulation of the splicing factor PTBP1 and its target, the embryonic pyruvate kinase isoform PKM2, is highly significant. PKM2 promotes the accumulation of glycolytic intermediates for macromolecule biosynthesis, a hallmark of the Warburg-like metabolism observed in highly proliferative, angiogenic ECs [10,13]. Furthermore, the simultaneous increase in LDHA ensures the rapid conversion of pyruvate to L(+)-lactate, regenerating NAD+ to sustain high glycolytic flux. The fact that VEGF silencing completely erased this complex metabolic signature confirms that mechanosensitive metabolic reprogramming in ECs is hierarchically governed by the AIBP–VEGF signaling node.

From a pathophysiological perspective, the mechanosensitive AIBP–-VEGF–-glycolysis axis offers a novel framework for understanding flow-related vascular diseases. As comprehensively highlighted by Zeng and colleagues in BIOCELL, the physical microenvironment and biomechanical forces continuously dictate cellular behavior, spatial organization, and fate decisions [29]. In regions of the vasculature exposed to uniform laminar shear stress, the mechanotransduction signaling and transient downregulation of AIBP might facilitate beneficial physiological remodeling or vascular repair. However, in regions characterized by disturbed or oscillatory flow such as arterial bifurcations heavily predisposed to atherosclerosis, the sustained dysregulation of mechanosensitive channels could lead to chronic AIBP depletion [4,5,6,7]. This would theoretically lock the endothelium in a state of persistent VEGF hyperactivation and glycolytic overdrive, promoting pathological angiogenesis, endothelial permeability, and leukocyte infiltration, which are critical steps in atherosclerotic plaque vulnerability and aneurysm progression. Consequently, stabilizing AIBP or therapeutically targeting downstream glycolytic nodes could represent a viable strategy to normalize endothelial metabolism in atheroprone regions.

Despite these mechanistic insights, several limitations of our study merit consideration. First, our investigations were exclusively conducted in vitro using HBMECs. While this model is robust for dissecting molecular epistasis, the microvascular endothelium of the brain possesses distinct barrier properties compared to the macrovascular endothelium (e.g., aortic or coronary arteries). Validating the AIBP–VEGF axis across diverse endothelial beds is necessary to confirm its generalizability. In addition to the inherent constraints of in vitro modeling, our current mechanistic findings are derived from a single microvascular endothelial model (HBMECs). Systematic validation across additional macrovascular (e.g., HAECs, HUVECs) and organ-specific microvascular beds will be required to confirm the universal applicability of this pathway. Second, while we utilized GsMTx4 to implicate mechanosensitive channels, the precise molecular identity of the channel, whether it be Piezo1, TRPV4, or a combination thereof remains to be pinpointed through specific genetic ablation. The precise molecular mechanisms by which AIBP senses or transduces shear stress signals warrant further investigation using protein–protein interaction assays, membrane microdomain analyses, and chromatin-based approaches. Third, in vivo animal models employing surgically induced disturbed flow (such as partial carotid artery ligation) are indispensable to fully ascertain the pathological relevance of AIBP-dependent mechano-metabolic coupling in the context of disease models like atherosclerosis.

## 5. Conclusions

**Figure 6:**
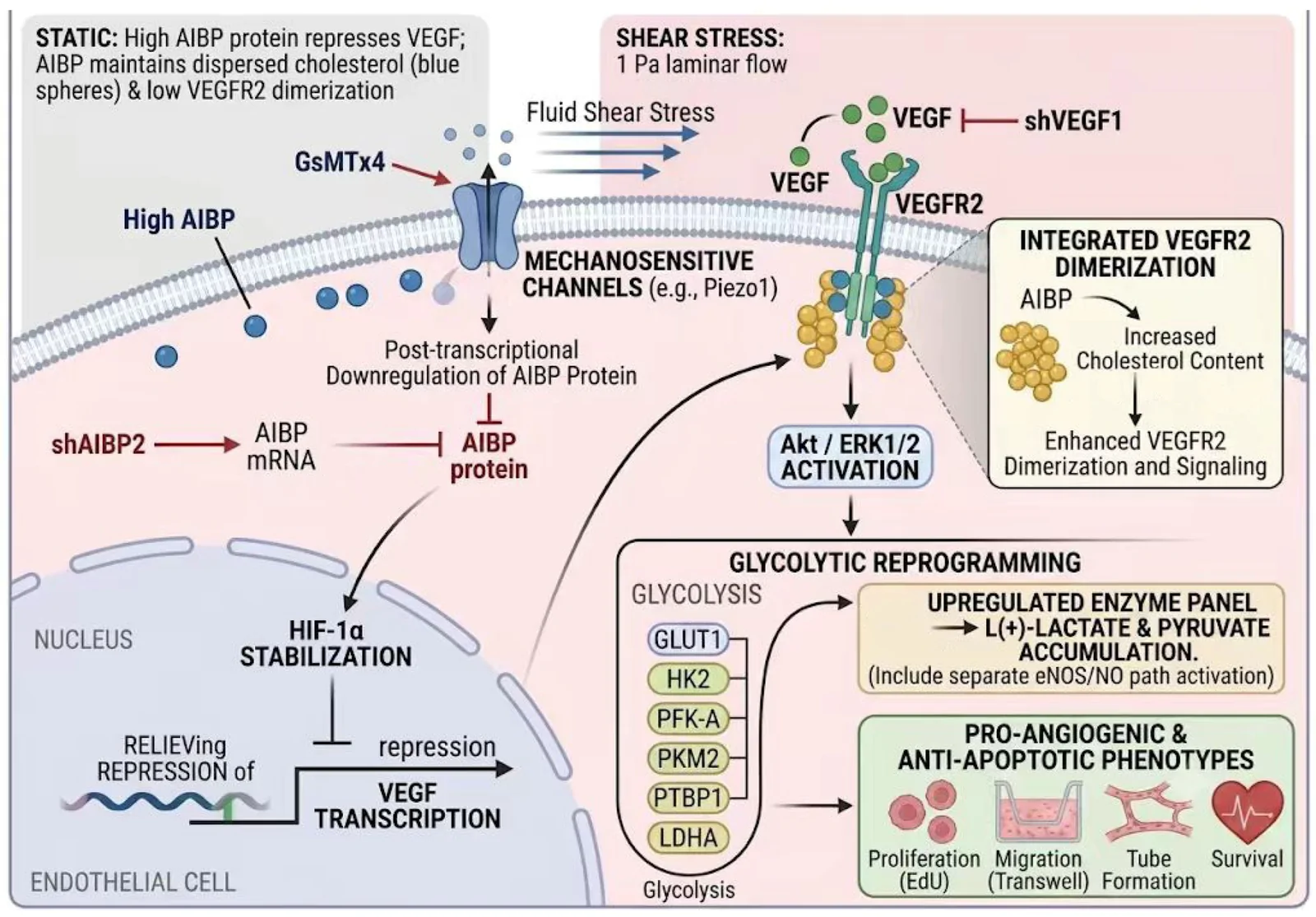
Proposed model of the shear stress/AIBP/VEGF/glycolysis signaling cascade in endothelial cells. Fluid shear stress activates membrane mechanosensitive channels (e.g., Piezo1), triggering AIBP downregulation and subsequent HIF-1α stabilization. This relieves basal repression of VEGF, activating downstream Akt and ERK1/2 phosphorylation and upregulating key glycolytic enzymes (GLUT1, HK2, PFK-A, PKM2, PTBP1, LDHA). The resulting glycolytic flux promotes endothelial proliferation, migration, and survival. Pharmacological inhibition with GsMTx4 preserves AIBP, while VEGF silencing confirms the absolute downstream requirement of VEGF in this pathway.

In conclusion, our study identifies a previously uncharted mechanotransduction pathway wherein fluid shear stress activates mechanosensitive channels to repress AIBP, thereby unleashing a VEGF-driven glycolytic shift that dictates endothelial activation. By delineating the AIBP—VEGF—glycolysis hierarchical axis (Figure 6), we provide novel molecular insights into how the endothelium precisely tunes its metabolic machinery in response to its physical environment, laying the groundwork for innovative therapeutic interventions in mechanobiology-related vascular disorders.

## Data Availability

Data supporting the findings of this study are available from the corresponding author upon reasonable request.

## Supplementary Materials

The following supporting information can be downloaded at: https://www.mdpi.com/article/10.3390/biology15181650/s1, Supplementary File S1: Supplementary Original Western Blot Images Note.
