# Supplementary material for: Fluid Shear Stress Drives Endothelial Glycolytic Reprogramming via an AIBP/VEGF Axis
Source: Biology (Basel). 2026 Sep 18;15(18):1650. doi: 10.3390/biology15181650 (PMC13604640; doi:10.3390/biology15181650)

Supplementary Original Western Blot Images Note:
The red arrows in the uncropped gel images indicate the specific target protein bands at their expected molecular weights.
These highlighted bands correspond exactly to the cropped representative images presented in the main manuscript figures.

For example:

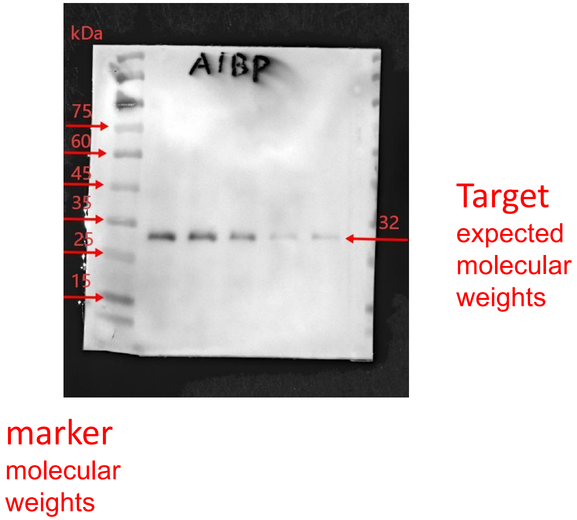

Fig1B

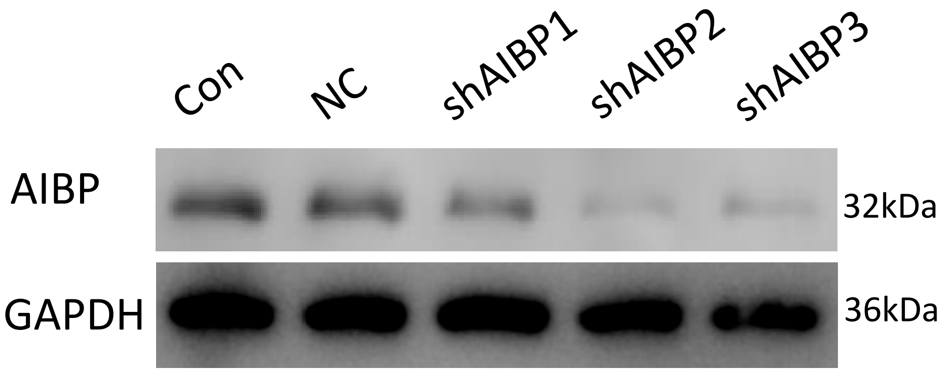

the representative band in Figure 1B corresponds to AIBP-1

GAPDH

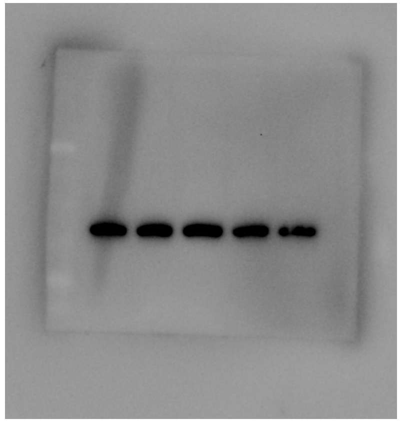

Uncropped gel for Figure 1B - GAPDH

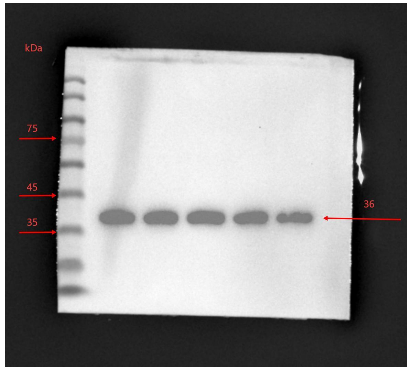

AIBP-1

Uncropped gel for Figure 1B - AIBP

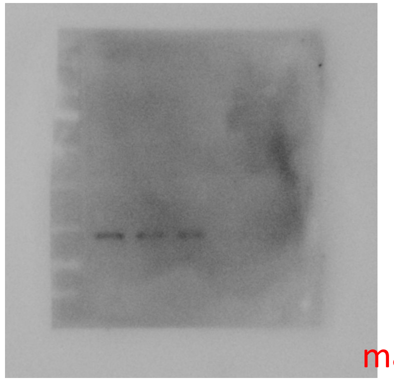

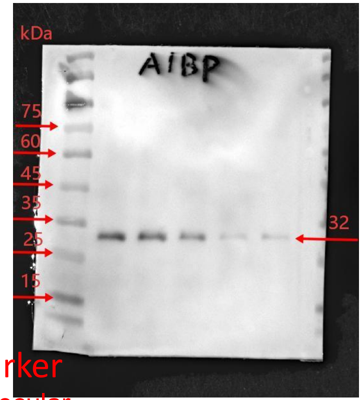

Target expected molecular weights

marker molecular weights

AIBP-2

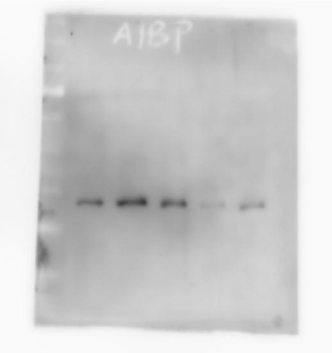

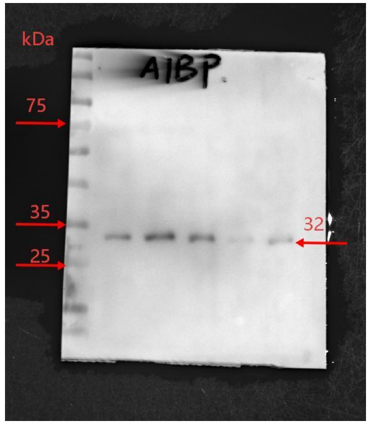

AIBP-3

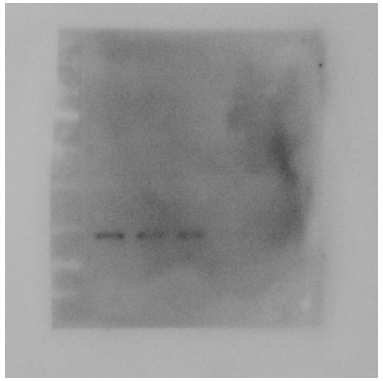

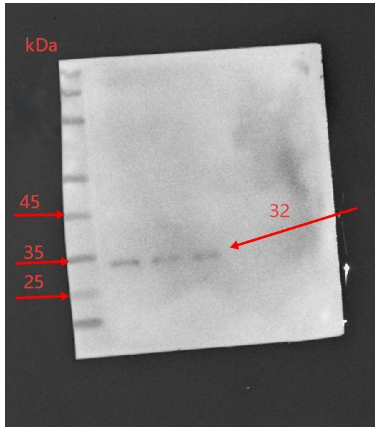

Fig1D

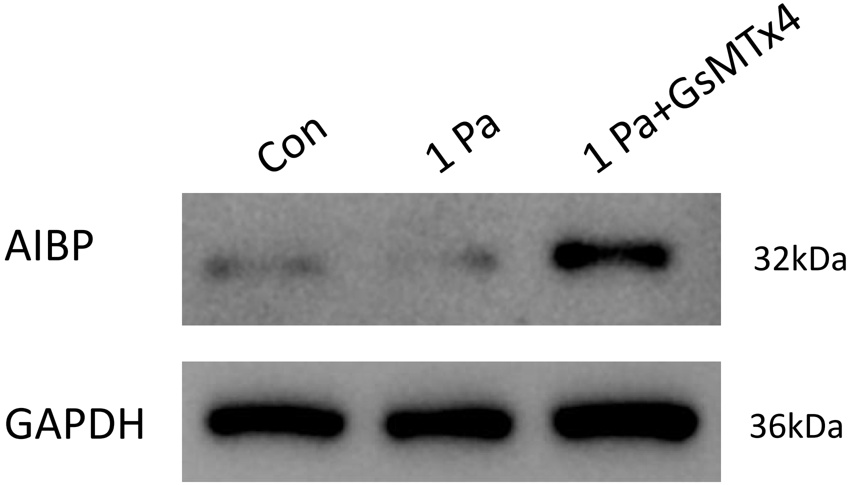

AIBP

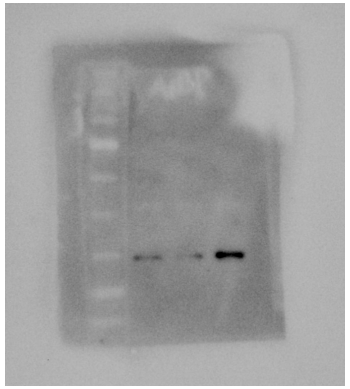

Uncropped gel for Figure 1D - AIBP

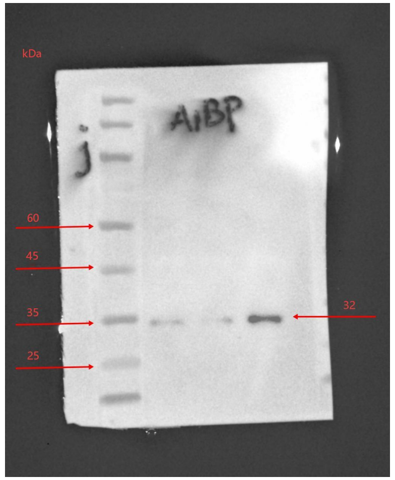

GAPDH

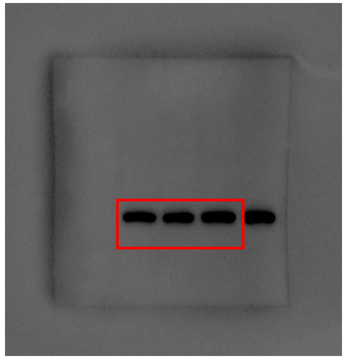

Uncropped gel for Figure 1D - GAPDH

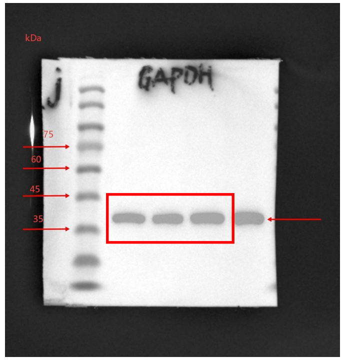

Fig1F

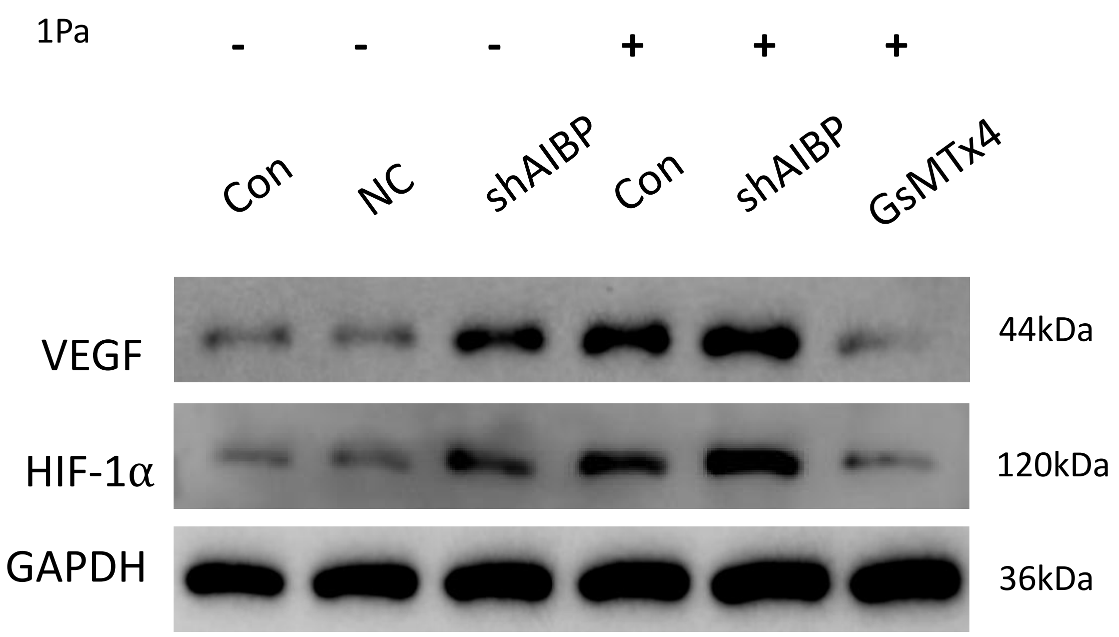

VEGF

Uncropped gel for Figure 1F - VEGF

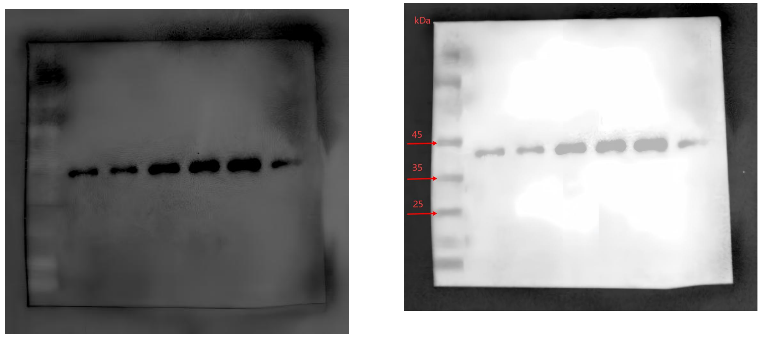

HIF-1α

Uncropped gel for Figure 1F HIF-1α

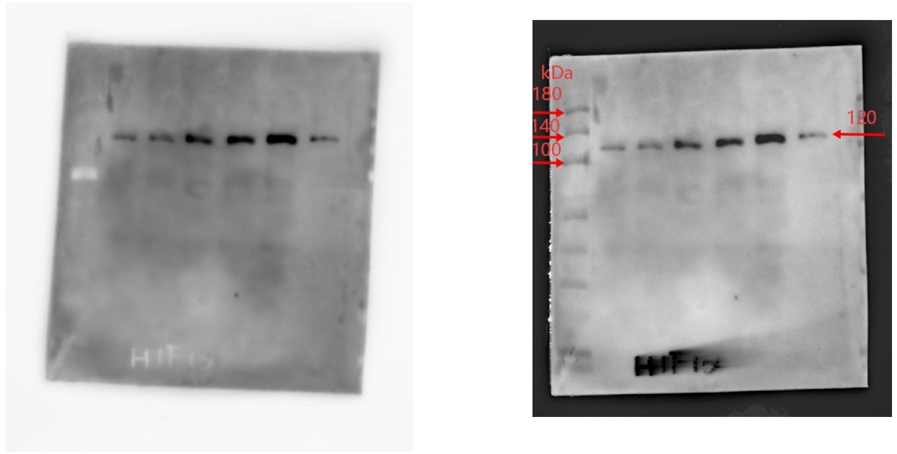

GAPDH

Uncropped gel for Figure 1F GAPDH

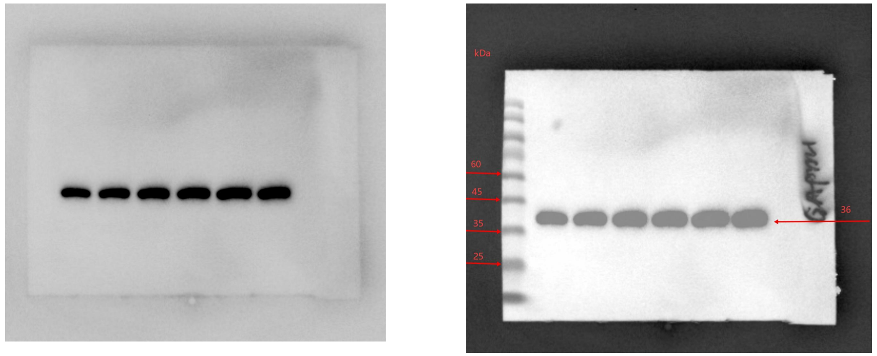

Fig3E

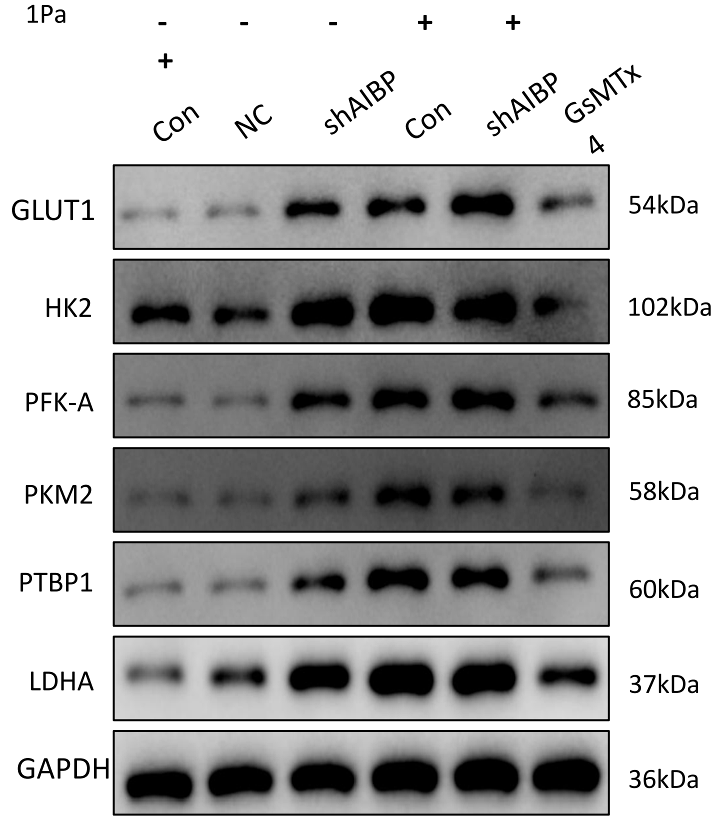

GLUT1 Uncropped gel for Figure 3E GLUT1

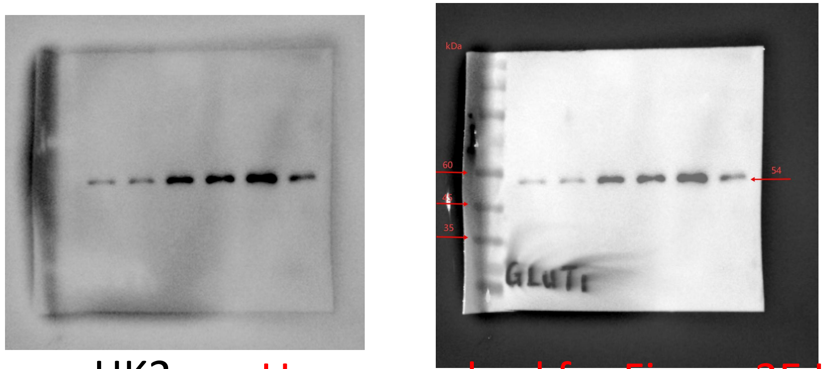

PKM2 Uncropped gel for Figure 3E PKM2

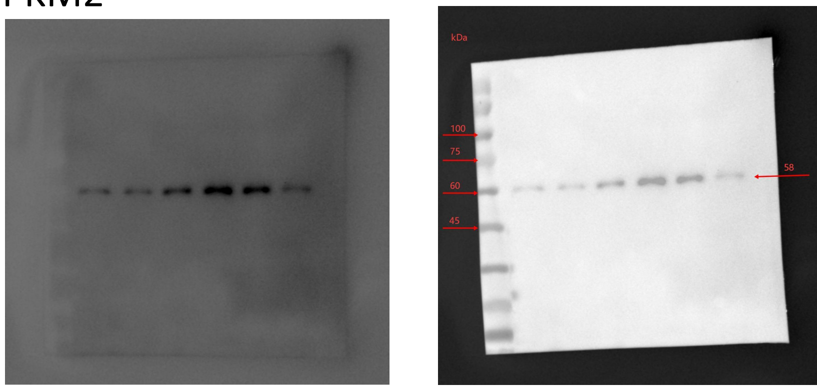

HK2 Uncropped gel for Figure 3E HK2

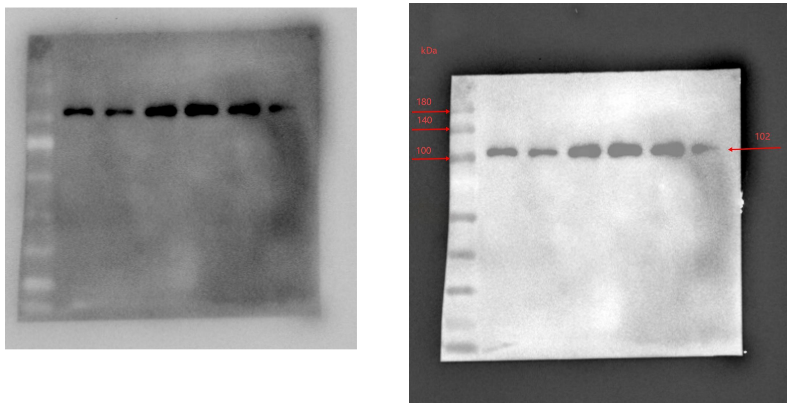

PTBP1 Uncropped gel for Figure 3E PTBP1

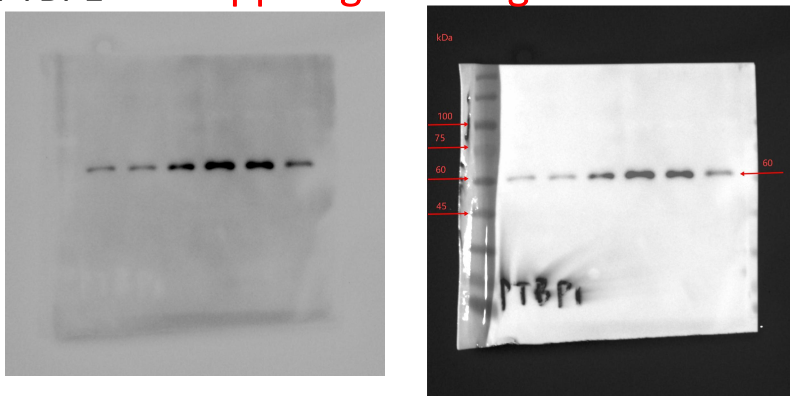

GAPDH Uncropped gel for Figure 3E GAPDH

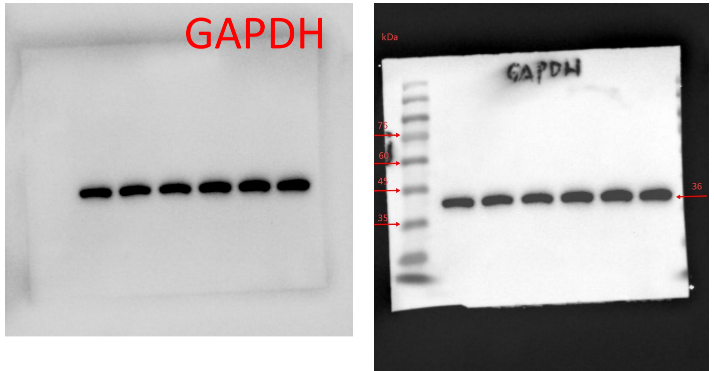

PFK-A Uncropped gel for Figure 3E PFK-A

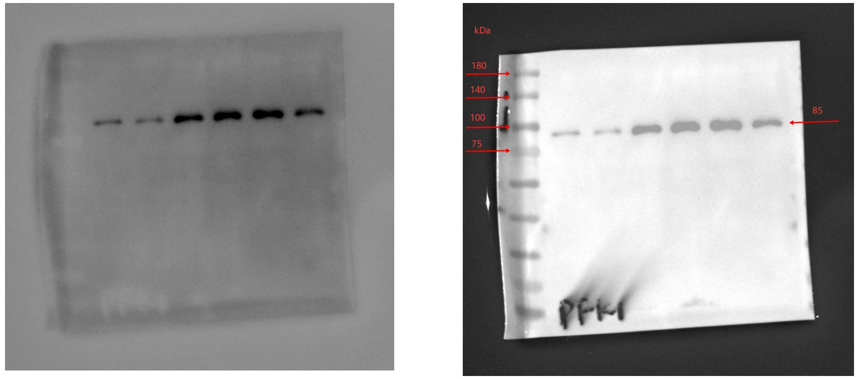

LDHA Uncropped gel for Figure 3E LDHA

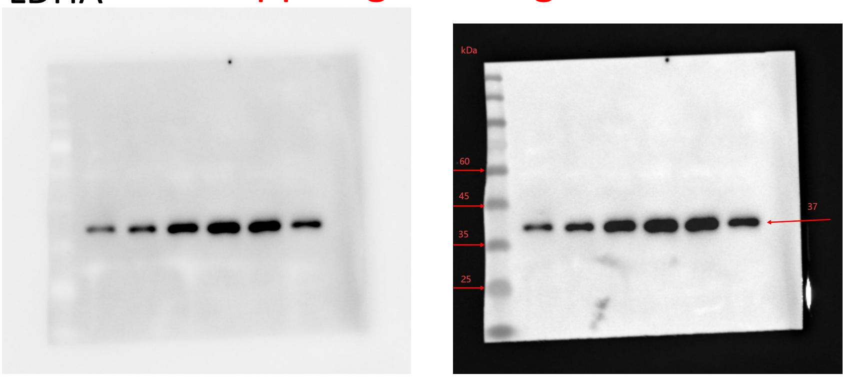

Fig3E

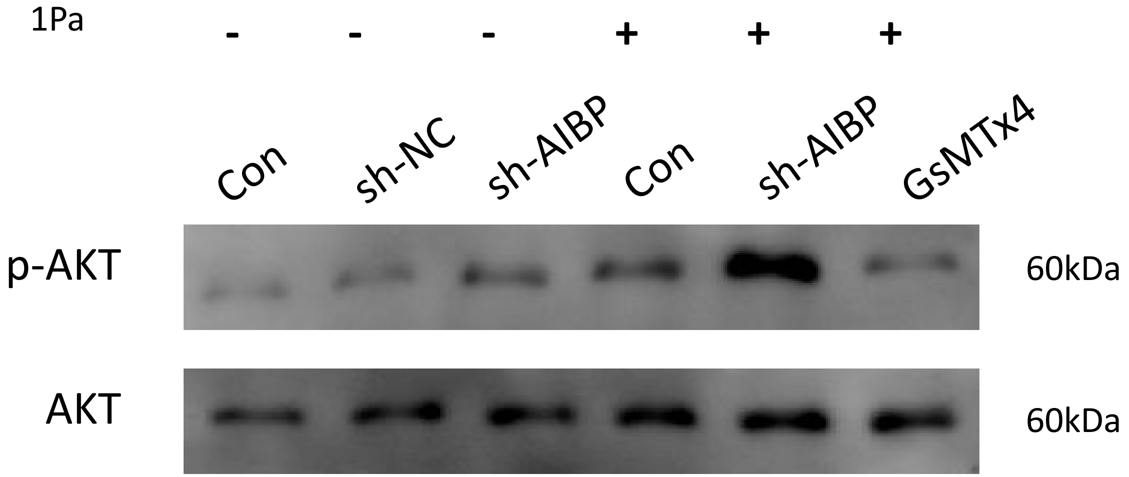

Uncropped gel for Figure 3E P-akt

p-AKT

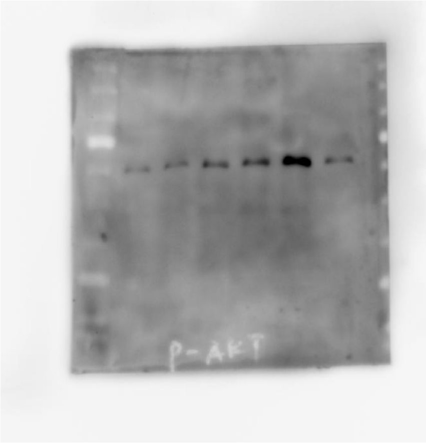

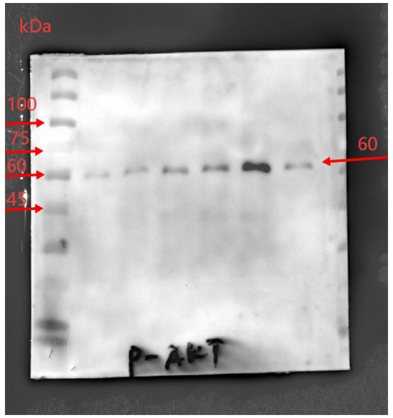

AKT

Uncropped gel for Figure 3E akt

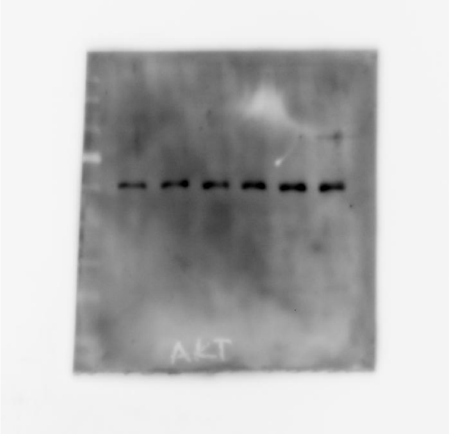

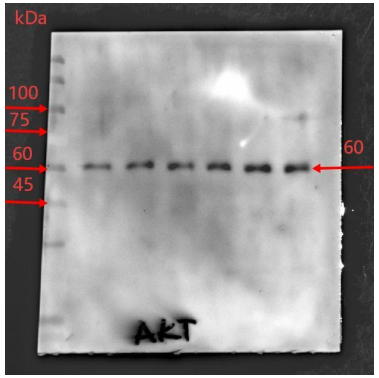

Fig3E

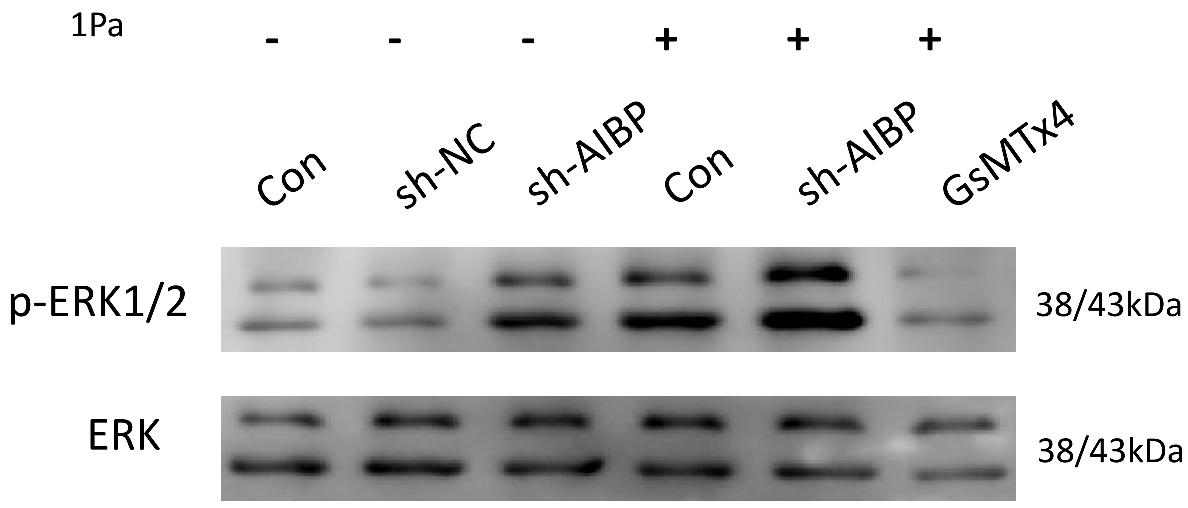

p-ERK1/2

Uncropped gel for Figure 3E p-Erk1/2

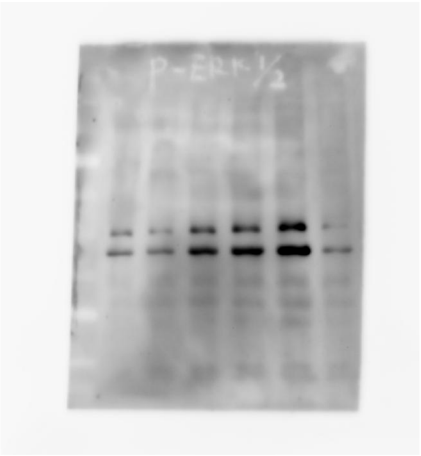

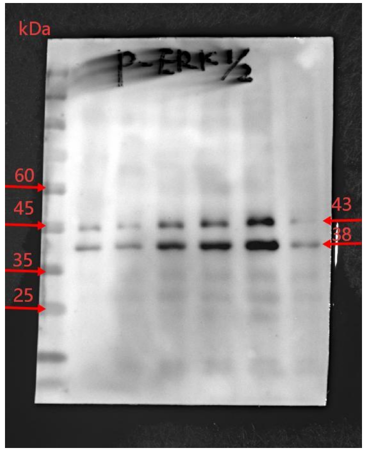

ERK

Uncropped gel for Figure 3E ERK

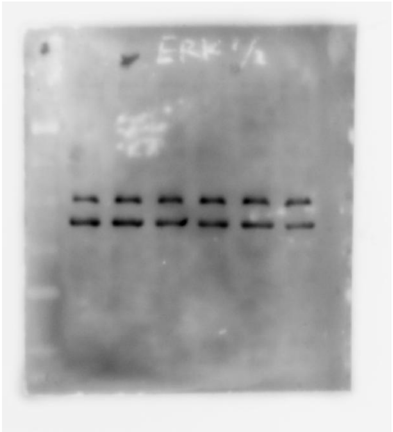

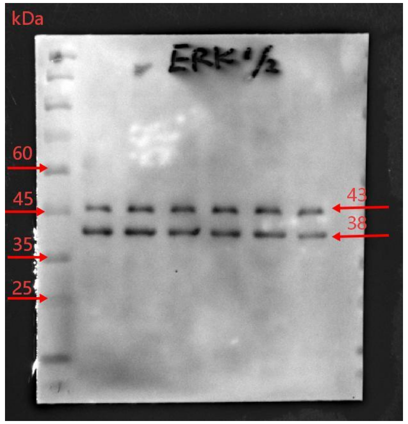

Fig4B

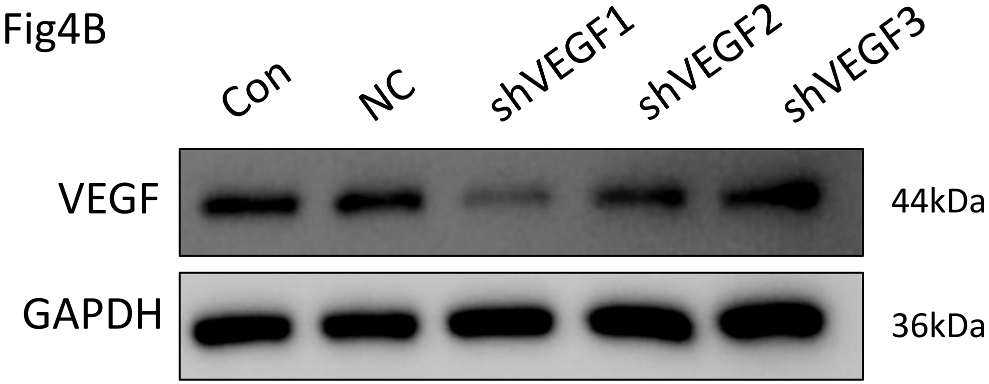

VEGF

Uncropped gel for Figure 4B VEGF

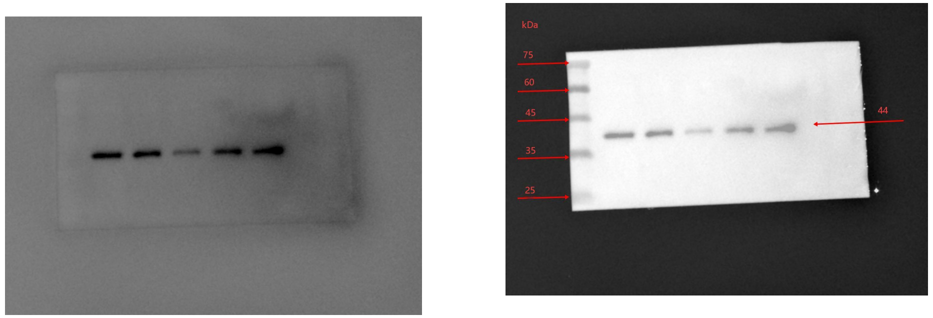

GAPDH

Uncropped gel for Figure 4B GAPDH

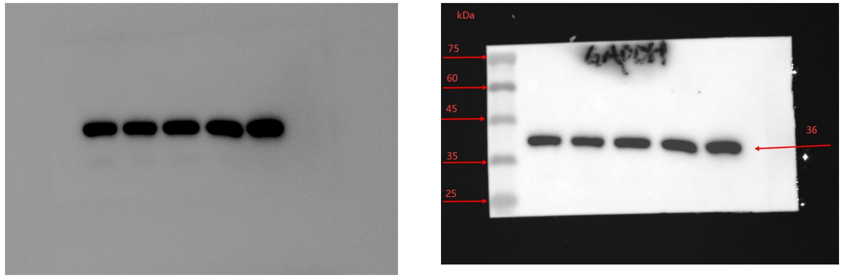

Fig4D

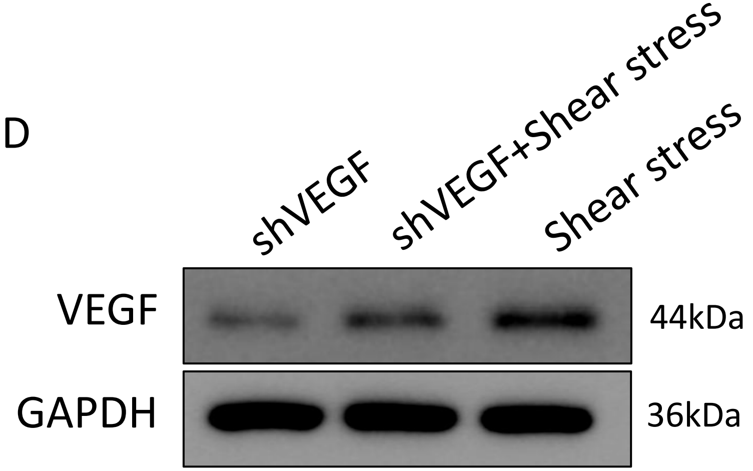

VEGF

Uncropped gel for Figure 4D VEGF

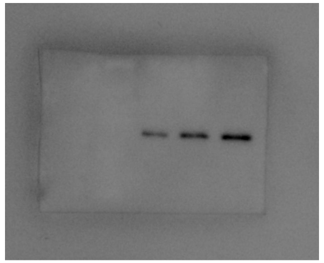

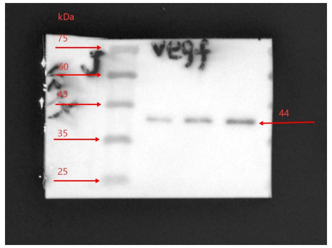

GAPDH

Uncropped gel for Figure 4D GAPDH

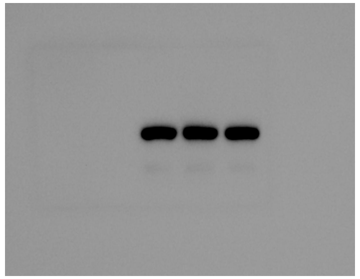

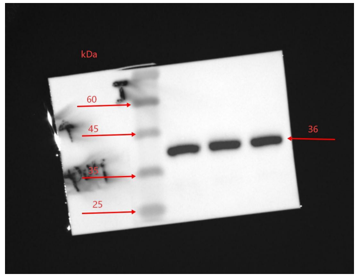

Fig5E

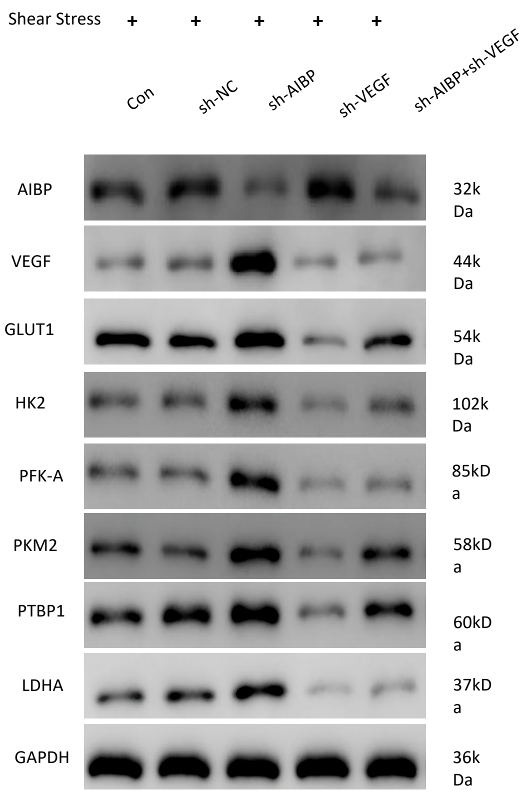

AIBP Uncropped gel for Figure 5E AIBP

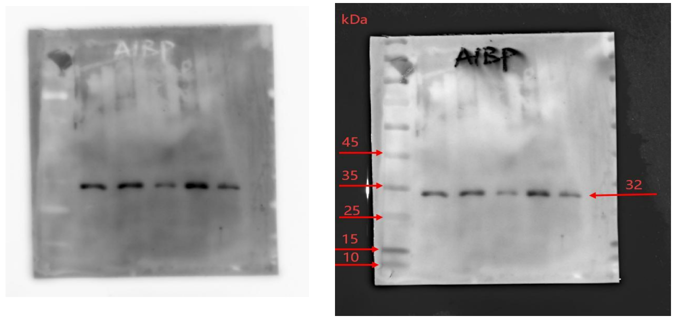

VEGF Uncropped gel for Figure 5E VEGF

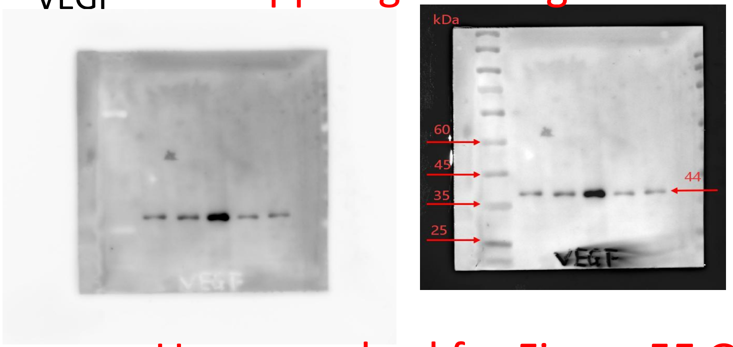

GLUT1 Uncropped gel for Figure 5E GLUT1

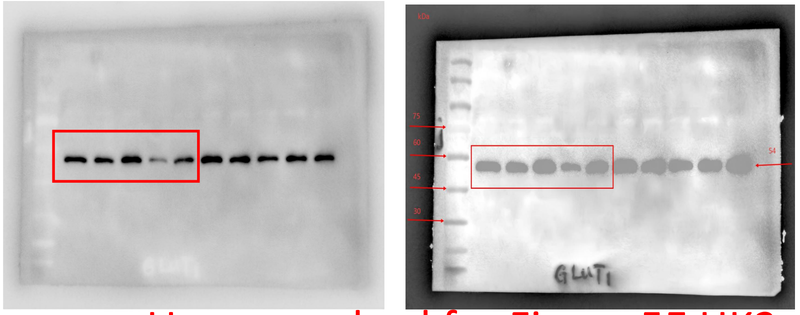

Uncropped gel for Figure 5E HK2

HK2

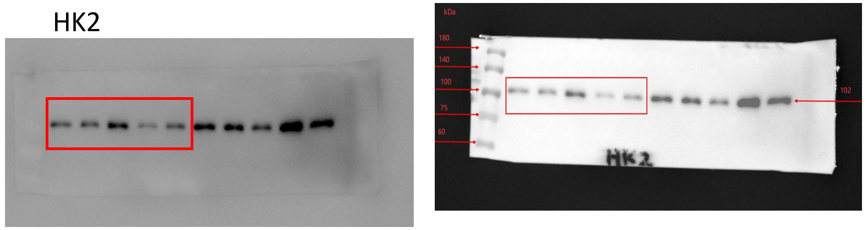

PFK-A Uncropped gel for Figure 5E PKF-A

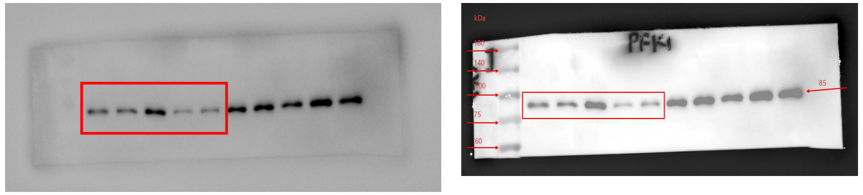

PKM2 Uncropped gel for Figure 5E PKM2

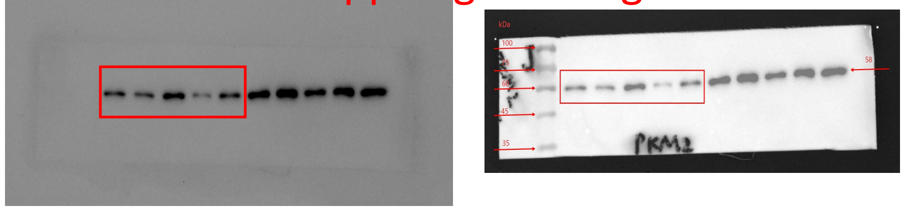

PTBP1 Uncropped gel for Figure 5E PTBP1

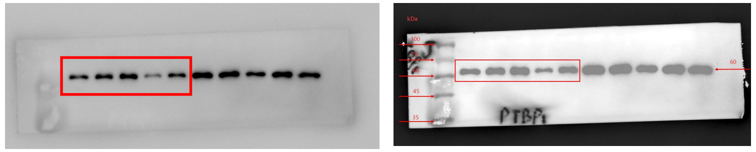

LDHA Uncropped gel for Figure 5E LDHA

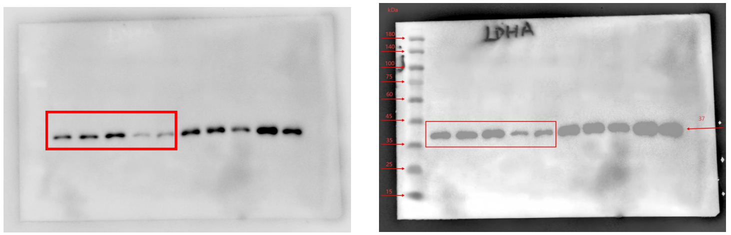

GAPDH Uncropped gel for Figure 5E GAPDH

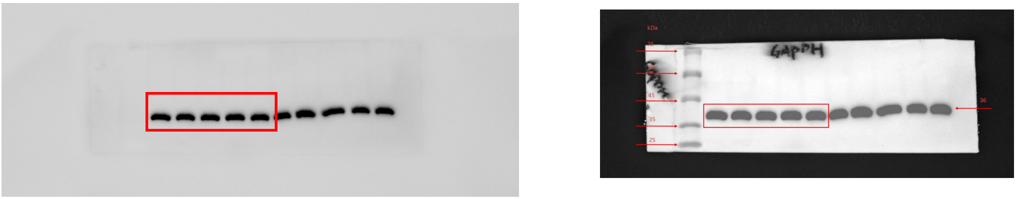

Supplement: Supplementary file 1 [file biology-15-01650-s001.zip › biology-4476602-supplementary.pdf]
